# A Genome-Wide Investigation of SNPs and CNVs in Schizophrenia

**DOI:** 10.1371/journal.pgen.1000373

**Published:** 2009-02-06

**Authors:** Anna C. Need, Dongliang Ge, Michael E. Weale, Jessica Maia, Sheng Feng, Erin L. Heinzen, Kevin V. Shianna, Woohyun Yoon, Dalia Kasperavičiūtė, Massimo Gennarelli, Warren J. Strittmatter, Cristian Bonvicini, Giuseppe Rossi, Karu Jayathilake, Philip A. Cola, Joseph P. McEvoy, Richard S. E. Keefe, Elizabeth M. C. Fisher, Pamela L. St. Jean, Ina Giegling, Annette M. Hartmann, Hans-Jürgen Möller, Andreas Ruppert, Gillian Fraser, Caroline Crombie, Lefkos T. Middleton, David St. Clair, Allen D. Roses, Pierandrea Muglia, Clyde Francks, Dan Rujescu, Herbert Y. Meltzer, David B. Goldstein

**Affiliations:** 1 Institute for Genome Sciences and Policy, Duke University, Durham, North Carolina, United States of America; 2 Department of Medical and Molecular Genetics, King's College London, Guy's Hospital, London, United Kingdom; 3 Department of Biostatistics and Bioinformatics, Duke University, Durham, North Carolina, United States of America; 4 Institute of Neurology, University College London, London, United Kingdom; 5 Genetic Unit, IRCCS San Giovanni di Dio Fatebenefratelli, Brescia, Italy; 6 Department of Biomedical Science and Biotech, University of Brescia, Brescia, Italy; 7 Division of Neurology, Department of Medicine, Duke University Medical Center, Durham, North Carolina, United States of America; 8 Psychiatric Unit, IRCCS San Giovanni di Dio Fatebenefratelli, Brescia, Italy; 9 Department of Psychiatry, Vanderbilt University School of Medicine, Nashville, Tennessee, United States of America; 10 University Hospitals Case Medical Center, Cleveland, Ohio, United States of America; 11 Department of Psychiatry and Behavioral Sciences, Duke University Medical Center, Durham, North Carolina, United States of America; 12 Genetics Division, GlaxoSmithKline, Research Triangle Park, North Carolina, United States of America; 13 Division of Molecular and Clinical Neurobiology, Department of Psychiatry, Ludwig-Maximilians-University, Munich, Germany; 14 Department of Psychiatry, Ludwig-Maximilians-University, Munich, Germany; 15 Genetics Research Centre GmbH (GRC), Munich, Germany; 16 Department of Mental Health, University of Aberdeen, Aberdeen, United Kingdom; 17 Division of Neuroscience and Mental Health, Neuroscience Laboratories, Burlington Danes, Hammersmith Hospital, London, United Kingdom; 18 Deane Drug Discovery Institute, Duke University Medical Center, Durham, North Carolina, United States of America; 19 Medical Genetics, GlaxoSmithKline R&D, Verona, Italy; Oxford Centre for Diabetes, Endocrinology, and Metabolism and Wellcome Trust Centre for Human Genetics, University of Oxford, United Kingdom

## Abstract

We report a genome-wide assessment of single nucleotide polymorphisms (SNPs) and copy number variants (CNVs) in schizophrenia. We investigated SNPs using 871 patients and 863 controls, following up the top hits in four independent cohorts comprising 1,460 patients and 12,995 controls, all of European origin. We found no genome-wide significant associations, nor could we provide support for any previously reported candidate gene or genome-wide associations. We went on to examine CNVs using a subset of 1,013 cases and 1,084 controls of European ancestry, and a further set of 60 cases and 64 controls of African ancestry. We found that eight cases and zero controls carried deletions greater than 2 Mb, of which two, at 8p22 and 16p13.11-p12.4, are newly reported here. A further evaluation of 1,378 controls identified no deletions greater than 2 Mb, suggesting a high prior probability of disease involvement when such deletions are observed in cases. We also provide further evidence for some smaller, previously reported, schizophrenia-associated CNVs, such as those in *NRXN1* and *APBA2*. We could not provide strong support for the hypothesis that schizophrenia patients have a significantly greater “load” of large (>100 kb), rare CNVs, nor could we find common CNVs that associate with schizophrenia. Finally, we did not provide support for the suggestion that schizophrenia-associated CNVs may preferentially disrupt genes in neurodevelopmental pathways. Collectively, these analyses provide the first integrated study of SNPs and CNVs in schizophrenia and support the emerging view that rare deleterious variants may be more important in schizophrenia predisposition than common polymorphisms. While our analyses do not suggest that implicated CNVs impinge on particular key pathways, we do support the contribution of specific genomic regions in schizophrenia, presumably due to recurrent mutation. On balance, these data suggest that very few schizophrenia patients share identical genomic causation, potentially complicating efforts to personalize treatment regimens.

## Author Summary

Schizophrenia is a highly heritable disease. While the drugs commonly used to treat schizophrenia offer important relief from some symptoms, other symptoms are not well treated, and the drugs cause serious adverse effects in many individuals. This has fueled intense interest over the years in identifying genetic contributors to schizophrenia. In this paper, we first show that common genetic variants, the focus of most research until recently, do not seem to have a major impact on schizophrenia predisposition. We then provide further evidence that very rare, large DNA deletions and duplications contribute to or explain a minority of schizophrenia cases. Although the small number of events identified here do not restrict focus to a finite set of molecular pathways, we do show one event that deletes a gene known to interact with *DISC1*, a gene known to cause psychiatric problems in one family. Such convergent findings have potential implications for the development of new therapies and patient subclassifications. We conclude that schizophrenia genetics research must turn sharply toward the identification of rare genetic contributors and that the most important tool in this effort will be complete whole-genome sequencing of patients whose clinical characteristics have been very thoroughly assessed.

## Introduction

Schizophrenia is a common neuropsychiatric disorder that is characterized by positive symptoms such as delusions, paranoia and hallucinations, negative symptoms including apathy, anhedonia, and social withdrawal, and extensive cognitive impairments that may have the greatest impact on overall function [1],[2]. While current antipsychotic drug treatments control positive symptoms in most patients, negative symptoms and cognitive impairments are much less improved by these agents [3]. A possible way to improve the treatment of schizophrenia is to identify genetic risk factors that might elucidate the underlying pathophysiological bases as well as help to subclassify patients at a molecular level in a manner helpful to therapy.

The etiology of schizophrenia as presently defined is not well understood. While there are clear environmental contributors to disease [4]–[10], it is clear that genetic predisposition is the major determinant of who develops schizophrenia, with heritability estimates as high as 80% [11],[12], placing schizophrenia amongst the most heritable of the common diseases.

Schizophrenia genetic research has traditionally focused on identifying linkage regions or on candidate genes and polymorphisms, such as the val158met polymorphism in the dopamine metabolizing gene *COMT*, or other types of variants such as VNTRs (*MAOA*, *DAT1*, *SLC6A4*). Such studies have implicated dozens of genes and variants, but none is generally accepted as definitively associated with schizophrenia [13]–[15].

It is now possible to represent the majority of common genetic variation by genotyping a selected set of tagging SNPs [16]. Such hypothesis-free genome-wide association studies allow the discovery of new genes and pathways affecting complex traits such as schizophrenia with much greater power to detect small effects than linkage studies. To date, there have been five genome-wide association studies (GWAS) of schizophrenia. The first study used a small sample size of 178 cases and 144 controls self-identifying as Caucasian and recruited in the US, and reported the association of a SNP in the pseudoautosomal region of the y chromosome at p = 3.7×10^−7^ [17]. The second used pooled DNA samples from 600 cases and 2,771 controls, all Ashkenazi Jews, and found no genome-wide significant association, although they reported a strong effect of a *RELN* SNP in females only [18]. The third used pooled DNA from 574 schizophrenia trios and 605 unaffected controls, all recruited in Bulgaria and again found no genome-wide significant association [19]. The next study of 738 cases and 733 controls (each about 30% African-American, 56% European American and 14% Other) found no evidence for the involvement of common SNPs in schizophrenia [14]. The most recent study included 479 cases compared to 2,937 WTCCC controls and replicated the top SNPs in two further datasets respectively comprising 1,664 cases and 3,541 controls and 6,666 cases and 7,897 controls [20]. Three of the loci remained associated after all analyses, one in *ZNF804A* and two in intergenic regions.

While the genotyping arrays used in genome-wide association studies have very limited capacity to detect the effects of rare single site variants, large copy number variants can be readily identified using these arrays, even if they occur in only one or a few subjects. Recently, considerable attention has turned towards identifying rare copy number variants that show elevated frequencies in various human diseases using these platforms.

In schizophrenia, four genome-wide screens for large CNVs have recently appeared. Two papers showed that large (>100 kb), rare deletions and duplications that disrupted genes were significantly more common in schizophrenia cases than controls [21],[22], and that the disrupted genes in patients were disproportionately from neurodevelopmental pathways [21]. Another showed that *de novo* CNVs were eight times more frequent in sporadic cases of schizophrenia than they were in familial cases or unaffected controls [23]. While neither of these papers succeeded in identifying particular CNVs as definitive schizophrenia risk factors, the greater load of CNVs reported in cases implicate this type of genetic variant in schizophrenia. Consistent with this, Stefansson *et al.* [24] recently screened for *de novo* CNVs and focused on three recurrent CNVs in 4,718 patients and 41,201 controls (including, for replication purposes only, all samples investigated in this study), located at 1q21.1, 15q11.2 and 15q13.3, with odds ratios of 14.8, 2.7 and 11.5. Two of these same loci were also reported as risk factors by the International Schizophrenia Consortium (also including the Aberdeen samples used here) [22].

These papers collectively suggest that the common disease-common variant hypothesis may be less relevant to schizophrenia than rare variants with highly penetrant effects [25]. However it should be noted that, to date, no WGA SNP study has been well powered to detect effects of common SNPs, since they have either been performed using pooled DNA, ethnically heterogeneous samples or small samples sizes, so it is not yet possible even to rule out reasonably large effects of common SNPs in schizophrenia. Additionally, despite these strong suggestions of a role for rare highly penetrant CNVs, there has been no test of whether any common CNVs also contribute to the risk of schizophrenia.

Here we investigated the effects of common SNPs, and both common and rare CNVs, on schizophrenia risk using genome-wide SNP data from the Illumina HumanHap genotyping BeadChips.

## Results

### Single Nucleotide Polymorphisms

We tested for SNP associations with schizophrenia with a logistic regression model using the PLINK software [26] and including sex and curated EIGENSTRAT axes as covariates. An additive genetic model was tested. A series of quality control procedures were undertaken before this analysis (for details see Text S1). The results were then annotated using the WGAViewer software [27]. No single polymorphism showed a genome-wide significant association in the discovery cohort. The top 100 associated SNPs are shown in Table 1. The most strongly associated SNPs at this stage were in the *ADAMTSL3* gene (lowest p = 1.34×10^−6^, Table 1).

**Table 1:** The top 100 SNPs associated with schizophrenia.

*Rank*	*SNP*	*Discovery P*	*Replication P*	*Combined P*	*OR*	*MAF Case*	*MAF Control*	*Chr*	*Position*	*SNP Type*	*Major/ Minor alleles*	*Closest gene*	*Distance to gene*
1	rs2135551	1.34E-06	0.03	1.35E-07	0.68	0.23	0.30	15	82498855	3PRIME UTR	A/G	*ADAMTSL3*	0
2	rs950169	2.28E-06	0.04	3.14E-07	0.68	0.23	0.30	15	82497465	NON SYNONYMOUS	C/T	*ADAMTSL3*	0
3	rs12910334	2.93E-06	0.21	2.87E-06	0.69	0.24	0.30	15	82749322	3PRIME UTR	G/A	*Q96PW8_HUMAN*	0
4	rs1911155	4.18E-06	N/A	4.18E-06	0.69	0.23	0.30	15	82578639	INTERGENIC	A/G	*ADAMTSL3*	79044
5	rs4814019	1.41E-05	0.77	9.39E-05	1.38	0.36	0.29	20	11456980	INTERGENIC	A/C	*C20orf61*	281900
6	rs4894485	1.90E-05	0.19	1.29E-05	1.37	0.43	0.35	3	176560380	INTRONIC	C/T	*NAALADL2*	0
7	rs1435194	1.92E-05	0.81	0.0001	1.44	0.25	0.19	5	105481256	INTERGENIC	C/A	*N/A*	N/A
8	rs2007744	1.96E-05	0.91	0.0002	1.52	0.18	0.13	9	78044132	INTRONIC	G/T	*PCSK5*	0
9	rs2717907	2.05E-05	0.70	0.0001	0.67	0.15	0.21	7	25269991	INTERGENIC	T/C	*NPVF*	−35361
10	rs715969	2.09E-05	0.52	6.28E-05	0.59	0.08	0.12	8	132543045	INTERGENIC	A/C	*ADCY8*	−419191
11	rs1146313	2.16E-05	0.88	0.0002	0.74	0.43	0.50	1	118827613	INTERGENIC	C/T	*SPAG17*	−298256
12	rs983037	2.19E-05	0.13	9.59E-06	0.73	0.32	0.39	2	12997671	INTERGENIC	A/C	*TRIB2*	197360
13	rs7175728	2.79E-05	0.55	8.55E-05	0.74	0.36	0.43	15	53681850	INTERGENIC	C/T	*PYGO1*	−13507
14	rs1229119	2.91E-05	N/A	2.91E-05	0.74	0.35	0.43	1	118825163	INTERGENIC	C/T	*SPAG17*	−295806
15	rs7943936	3.10E-05	0.29	3.61E-05	1.34	0.53	0.46	11	130214277	INTERGENIC	C/T	*SNX19*	36708
16	rs1463259	3.17E-05	N/A	3.17E-05	0.70	0.22	0.28	8	82900300	INTRONIC	C/T	*SNX16*	0
17	rs725710	3.30E-05	0.41	6.18E-05	0.65	0.11	0.16	16	73431014	INTERGENIC	C/A	*WDR59*	33961
18	rs7379110	3.41E-05	0.23	2.80E-05	0.74	0.33	0.39	5	34089378	INTRONIC	C/T	*C1QTNF3*	0
19	rs7943757	3.64E-05	0.55	0.0001	0.74	0.43	0.50	11	130221367	INTERGENIC	C/T	*SNX19*	29618
20	rs2239385	3.96E-05	1.00	0.0004	1.39	0.29	0.23	21	39949617	INTRONIC	A/G	*B3GALT5*	0
21	rs505703	4.18E-05	0.50	0.0001	1.49	0.19	0.13	4	13530080	INTERGENIC	C/T	*FAM44A*	−291654
22	rs230669	4.28E-05	0.93	0.0003	0.74	0.34	0.42	11	77553034	INTERGENIC	A/G	*KCTD21*	6917
23	rs1943624	4.47E-05	0.48	0.0001	0.75	0.46	0.53	11	-9	N/A	C/T	*N/A*	N/A
24	rs4772445	4.88E-05	N/A	4.88E-05	1.52	0.17	0.12	13	101601632	INTRONIC	G/A	*FGF14*	0
25	rs4714675	5.52E-05	1.00	0.0005	0.63	0.09	0.13	6	43395871	REGULATORY REGION	C/T	*CRIP3*	−11358
26	rs3748376	5.64E-05	N/A	5.64E-05	0.72	0.23	0.29	15	83129356	INTRONIC	C/T	*ZNF592*	0
27	rs6696438	5.86E-05	0.42	0.0001	0.64	0.09	0.13	1	194351115	INTERGENIC	C/T	*KCNT2*	110421
28	rs2000191	6.68E-05	N/A	6.68E-05	1.93	0.07	0.04	6	22612727	INTERGENIC	A/C	*HDGFL1*	−64930
29	rs7119425	6.71E-05	0.19	4.03E-05	0.75	0.40	0.46	11	130238395	INTERGENIC	C/T	*SNX19*	12590
30	rs7676721	6.76E-05	0.38	0.0001	1.39	0.28	0.22	4	13525449	INTERGENIC	G/T	*FAM44A*	−287023
31	rs3786603	7.05E-05	0.53	0.0002	1.87	0.07	0.04	19	16596788	INTRONIC	C/T	*MED26*	0
32	rs1586030	7.07E-05	N/A	7.07E-05	0.75	0.41	0.47	8	3496385	INTERGENIC	C/T	*CSMD1*	−237389
33	rs4745431	7.26E-05	0.02	3.83E-06	0.75	0.40	0.45	9	77461915	INTERGENIC	C/T	*PCSK5*	−233491
34	rs9375543	7.75E-05	0.52	0.0002	0.75	0.37	0.44	6	128352833	INTRONIC	T/C	*PTPRK*	0
35	rs2631879	8.26E-05	0.16	3.81E-05	1.55	0.15	0.10	8	21174982	INTERGENIC	C/T	*GFRA2*	418830
36	rs12365680	8.65E-05	0.88	0.0005	0.76	0.38	0.44	11	130254269	INTRONIC	G/A	*SNX19*	0
37	rs6737733	8.91E-05	0.94	0.0006	0.74	0.28	0.34	2	118291938	INTRONIC	C/T	*DDX18*	0
38	rs2216670	0.0001	0.72	0.0004	1.81	0.07	0.04	19	16555094	INTRONIC	T/G	*MED26*	0
39	rs154981	0.0001	N/A	1.00E-04	1.32	0.53	0.46	6	32988971	INTERGENIC	T/C	*NM 002118*	21413
40	rs566353	0.0001	N/A	1.00E-04	1.32	0.48	0.43	11	60472288	INTRONIC	C/T	*SLC15A3*	0
41	rs3021461	0.0001	0.28	9.40E-05	0.71	0.19	0.24	3	129578342	INTRONIC	A/G	*EEFSEC*	0
42	rs2844511	0.0001	N/A	1.00E-04	0.76	0.42	0.50	6	31497763	INTERGENIC	C/T	*Q5SS58_HUMAN*	5768
43	rs9402011	0.0001	N/A	1.00E-04	0.73	0.25	0.31	6	128347581	INTRONIC	T/C	*PTPRK*	0
44	rs4280783	0.0001	N/A	1.00E-04	0.76	0.38	0.44	4	67771960	INTERGENIC	C/T	*CENPC1*	248624
45	rs2304066	0.0001	N/A	1.00E-04	1.39	0.24	0.19	5	145760885	INTERGENIC	T/C	*TCERG1*	−46181
46	rs1379552	0.0001	N/A	1.00E-04	0.72	0.20	0.25	5	111007057	INTERGENIC	T/C	*C5orf13*	85351
47	rs12680924	0.0001	0.70	0.0004	1.31	0.48	0.43	8	15098795	INTRONIC	G/A	*SGCZ*	0
48	rs1328657	0.0001	N/A	1.00E-04	1.33	0.32	0.27	13	96981887	INTERGENIC	C/T	*RAP2A*	63644
49	rs718796	0.0001	N/A	1.00E-04	1.75	0.08	0.05	7	16585507	INTRONIC	A/G	*ANKMY2*	20425
50	rs17257972	0.0001	0.67	0.0003	1.31	0.52	0.46	1	118908135	INTERGENIC	G/A	*TBX15*	319054
51	rs4669887	0.0001	0.48	0.0002	1.38	0.26	0.20	2	12893630	INTERGENIC	G/T	*TRIB2*	93319
52	rs6868716	0.0001	N/A	1.00E-04	1.54	0.12	0.09	5	145769452	INTERGENIC	C/T	*TCERG1*	−37614
53	rs7762279	0.0001	N/A	1.00E-04	0.63	0.08	0.12	6	32863268	INTERGENIC	T/C	*A2ADX3_HUMAN*	−23981
54	rs596958	0.0001	0.40	0.0002	1.81	0.07	0.04	21	39945361	INTRONIC	G/A	*B3GALT5*	0
55	rs764855	0.0001	N/A	1.00E-04	1.31	0.46	0.40	11	56366988	INTERGENIC	G/T	*Q3C1V7_HUMAN*	−23228
56	rs7815272	0.0001	N/A	1.00E-04	0.73	0.23	0.29	8	82854364	INTERGENIC	G/T	*SNX16*	20013
57	rs7775397	0.0002	N/A	2.00E-04	0.64	0.09	0.13	6	32369230	NON SYNONYMOUS	T/G	*C6orf10*	0
58	rs2523554	0.0002	N/A	2.00E-04	0.76	0.36	0.42	6	31439808	INTERGENIC	T/C	*HLA-B*	−6894
59	rs1766803	0.0002	N/A	2.00E-04	0.76	0.35	0.41	1	119185341	INTERGENIC	T/C	*TBX15*	41848
60	rs3934902	0.0002	0.12	6.26E-05	0.72	0.17	0.23	9	85367221	INTERGENIC	G/A	*FRMD3*	−23940
61	rs1217461	0.0002	N/A	2.00E-04	0.73	0.20	0.24	5	104241987	INTERGENIC	T/G	*N/A*	N/A
62	rs1289726	0.0002	N/A	2.00E-04	0.63	0.07	0.11	1	162679471	INTERGENIC	G/T	*PBX1*	−116213
63	rs4329498	0.0002	N/A	2.00E-04	1.30	0.52	0.46	1	118935132	INTERGENIC	T/C	*TBX15*	292057
64	rs9427727	0.0002	0.18	9.77E-05	1.32	0.40	0.34	1	200305568	INTERGENIC	C/T	*GPR37L1*	−53084
65	rs1054869	0.0002	0.12	6.34E-05	0.77	0.43	0.49	11	130247840	DOWNSTREAM	G/A	*SNX19*	3145
66	rs7758512	0.0002	N/A	2.00E-04	1.54	0.13	0.09	6	30078568	INTRONIC	T/G	*Q6ZU40_HUMAN*	0
67	rs6924102	0.0002	N/A	2.00E-04	0.76	0.40	0.47	6	32919361	INTRONIC	A/G	*PSMB8*	0
68	rs4236064	0.0002	N/A	2.00E-04	1.41	0.21	0.16	6	39522005	INTRONIC	G/T	*KIF6*	0
69	rs4487082	0.0002	0.01	5.70E-06	0.59	0.06	0.09	2	229432205	INTERGENIC	A/G	*PID1*	164729
70	rs2119137	0.0002	N/A	2.00E-04	1.31	0.40	0.34	2	174725960	INTRONIC	A/G	*OLA1*	0
71	rs39829	0.0002	N/A	2.00E-04	0.76	0.39	0.44	5	13772997	INTRONIC	A/C	*DNAH5*	0
72	rs11635597	0.0002	N/A	2.00E-04	0.74	0.24	0.29	15	82966703	3PRIME UTR	C/T	*ZSCAN2*	0
73	rs16878312	0.0002	N/A	2.00E-04	0.75	0.28	0.34	5	71741140	INTERGENIC	C/A	*ZNF366*	33850
74	rs7603333	0.0002	N/A	2.00E-04	1.30	0.49	0.43	2	79211103	INTERGENIC	G/A	*Q53S57_HUMAN*	6855
75	rs2631878	0.0002	0.39	0.0003	1.52	0.14	0.10	8	21173339	INTERGENIC	G/A	*GFRA2*	420473
76	rs4745430	0.0002	0.01	8.30E-06	0.76	0.33	0.39	9	77461845	INTERGENIC	T/C	*PCSK5*	−233561
77	rs4901053	0.0002	N/A	2.00E-04	0.77	0.46	0.52	14	50287324	INTRONIC	T/G	*NIN*	0
78	rs5756219	0.0002	N/A	2.00E-04	1.30	0.49	0.41	22	35221804	INTRONIC	A/C	*EIF3D*	0
79	rs220420	0.0002	N/A	2.00E-04	0.76	0.33	0.38	6	86918598	INTERGENIC	G/T	*C6orf161*	−416093
80	rs1294028	0.0002	0.69	0.0006	1.31	0.42	0.36	1	9287221	INTRONIC	G/A	*SPSB1*	0
81	rs7738388	0.0002	N/A	2.00E-04	1.34	0.32	0.27	6	141494838	INTERGENIC	T/C	*N/A*	N/A
82	rs12966353	0.0002	0.56	0.0005	1.31	0.39	0.33	18	24297001	INTERGENIC	C/A	*CDH2*	−285812
83	rs2236711	0.0002	N/A	2.00E-04	0.77	0.43	0.49	11	130244494	INTERGENIC	G/A	*SNX19*	6491
84	rs927743	0.0002	0.22	0.0001	1.31	0.46	0.39	1	59141535	INTERGENIC	G/T	*JUN*	−118948
85	rs2073848	0.0002	N/A	2.00E-04	1.43	0.19	0.14	11	113557114	INTRONIC	T/C	*ZBTB16*	0
86	rs6457374	0.0002	N/A	2.00E-04	0.74	0.24	0.30	6	31380240	INTERGENIC	T/C	*1C07_HUMAN*	−32354
87	rs2372897	0.0002	N/A	2.00E-04	0.76	0.36	0.43	11	77420474	INTERGENIC	A/G	*KCTD14*	−8486
88	rs2071538	0.0002	N/A	2.00E-04	1.37	0.26	0.20	6	32926656	INTRONIC	G/A	*TAP1*	0
89	rs1482294	0.0002	N/A	2.00E-04	1.30	0.46	0.39	12	128276744	INTRONIC	G/A	*TMEM132D*	0
90	rs3099844	0.0002	N/A	2.00E-04	0.67	0.10	0.14	6	31556955	INTERGENIC	G/T	*HCP5*	15495
91	rs1794282	0.0002	N/A	2.00E-04	0.65	0.08	0.13	6	32774504	INTERGENIC	C/T	*HB25_HUMAN*	−20208
92	rs8023192	0.0002	N/A	2.00E-04	1.30	0.49	0.44	14	20265311	INTERGENIC	C/A	*FAM12A*	−18628
93	rs1451487	0.0002	N/A	2.00E-04	0.71	0.15	0.19	2	199703113	INTERGENIC	T/C	*SATB2*	139355
94	rs10516269	0.0002	N/A	2.00E-04	1.41	0.20	0.14	4	13546358	INTERGENIC	T/G	*FAM44A*	−307932
95	rs30168	0.0003	N/A	3.00E-04	0.77	0.38	0.44	5	13772089	NON SYNONYMOUS	G/A	*DNAH5*	0
96	rs4800613	0.0003	N/A	3.00E-04	0.76	0.29	0.34	18	20861721	INTERGENIC	A/G	*ZNF521*	34168
97	rs3903663	0.0003	N/A	3.00E-04	0.74	0.25	0.30	6	128354781	INTRONIC	A/G	*PTPRK*	0
98	rs4658504	0.0003	0.70	0.0009	0.77	0.34	0.40	1	241107670	INTERGENIC	C/T	*CEP170*	246683
99	rs3131379	0.0003	N/A	3.00E-04	0.66	0.09	0.13	6	31829012	INTRONIC	G/A	*MSH5*	0
100	rs1377347	0.0003	N/A	3.00E-04	1.33	0.33	0.27	4	41067359	INTRONIC	A/C	*LIMCH1*	0

Displayed are: (1) rs# for the top 100 SNPs; (2) combined *p* value in Munich and Aberdeen discovery cohorts; (3) *p* value in the second independent cohort; (4) Combined *p* value in discovery and replication cohorts using Stouffer's method [60]; (5) odds ratio; (6) minor allele frequency in patients; (7) minor allele frequency in controls; (8) chromosome; (9) chromosomal position; (10) a description of the relative position of the SNP in the closest gene; (11) major and minor alleles; (12) the symbol of the closest gene; and (13) distance to the closest gene.

Following these analyses, we genotyped the top 100 polymorphisms in a further independent Munich cohort of 298 schizophrenic patients and 713 healthy controls, all self-identifying as of German or central European ancestry. Using the Sequenom iPLEX system, we successfully genotyped 98 of the 100 SNPs and found that 8 of these 98 variants showed an association that was significant at the 0.05 level in the independent cohort (rs2135551, rs950169, rs1911155, rs4745431, rs4745430, rs4487082, rs3748376 and rs11635597). These included the most strongly associated three SNPs in the list: rs2135551, rs950169 and rs1911155 in *ADAMTSL3* (in linkage disequilibrium with one another) (Table 1). Since 3 of these 8 SNPs are in strong LD, this is approximately the number of significant associations we would expect by chance at p<0.05, however in all 8 cases the direction of effect was the same as in the original cohort. The combined p value for the strongest associated SNP (rs2135551) across the original and first replication studies is 1.3×10^−7^. If we use a Bonferroni correction for all the SNPs considered in this study (312,565 SNPs that passed quality control and the minor allele restriction), the 0.05 experiment-wide cut-off is 0.05/312565 = 1.6×10^−7^, which means that this association is suggestive, but falls short of the proposed threshold for genome-wide significance of <5×10^−8^ [28].

The most associated polymorphism (rs2135551) is in the 3′UTR of exon 30 of *ADAMTSL3*. To investigate a possible functional mechanism for this SNP, we tested for association with alternative splicing events in the associated region (exons 28, 29, and 30) using brain tissue. We found that the associated SNPs rs950169 and rs2135551 showed a highly significant correlation with the use of an alternative splice acceptor site, resulting in a truncated PLAC (protease and lacunin) domain in the *ADAMTSL3* protein (p<0.0001, Figure S2). We then confirmed a causal relationship between rs950169 and the observed splicing pattern using a MINIGENE system (Text S1), and showed an association between rs950169 genotype and the splice form of *ADAMTSL3* in brain tissue from both healthy controls and Alzheimer's disease patients (Figure S3). Finally, we showed that *ADAMTSL3* is particularly strongly expressed in hippocampal pyramidal cells (Figure S4).

To try to further confirm this association, we genotyped the *ADAMTSL3* SNP rs2135551 (and, by proxy, rs950169) using TaqMan assays in a further 394 cases and 524 controls from Italy. However, in this cohort the p value was 0.311. We then investigated the SNP in 589 schizophrenia cases and 11,491 controls from Iceland and found that it did not associate with schizophrenia in these subjects either (p = 0.12) (Hreinn Stefánsson, personal communication). Finally, we failed to replicate this association in a third cohort of 179 cases and 267 controls of European-American ancestry genotyped using the Illumina-610 Quad genotyping chip (and passing through the same quality control procedures used for the discovery cohorts). The p value for rs213551 was 0.19 and for rs950169 it was 0.22. Since we had whole-genome data for the European-American cohort, we also checked the top 100 SNPs from the discovery cohort but none of the SNPs associated after being corrected for 100 tests (lowest raw p value = 0.02).

In the combined Munich and Aberdeen discovery cohort, the MAF of rs2135551 was 0.23 in cases and 0.30 in controls. In the Italian cohort, the MAF was a little higher at 0.33 in cases and 0.36 in controls, in Iceland the MAF was 0.25 in cases and 0.27 in controls, and in the European-American sample it was 0.28 in cases and 0.25 in controls. These data indicate that despite its real functional effect, the top association with schizophrenia in the discovery and first replication cohorts is likely to be a false positive.

To assess more formally the combined evidence of association for the ADAMTSL3 SNPS we extended the Bayesian framework developed by Wakefield [29] to consider the cumulative posterior odds for the hypothesis of true association with schizophrenia, as data from successive datasets are added (see Methods). We found that the posterior odds for true association at rs2135551 tracked from 0.10 (after GWAS) to 0.68 (after Replication 1 in Germany) to 0.30 (after Replication 2 in Italy) to 0.15 (after Replication 3 in deCODE) to 0.02 (after Replication 4 in the US cohort). Thus, under these assumptions, the odds for the association being true never rise above one, and finally reduce to the null hypothesis being 50 times more likely than the alternative hypothesis of true association.

To investigate the degree of weight to put on the functional effect of rs950169, using data from a genome-wide association study examining the effects of approximately 550,000 SNPs on expression of 1.41 million exons in human frontal cortex tissue (Text S1), we looked to see what percentage of exons show association with a nearby (+/− 100 kb) SNP at or below the level of significance that rs950169 associates with the expression of the *ADAMTSL3* exon 3605495 (https://www.affymetrix.com/analysis/netaffx/index.affx). We found that 84,840, or 6%, of exons showed a SNP association of this magnitude. This illustrates the importance of weighing functional evidence against an appropriate null hypothesis, and we note that in many cases arriving at a quantitative evaluation of this kind can be very difficult. This fact, along with the failed replication in the Italian, Icelandic and US data, suggests that evidence of functional effect for SNPs implicated in GWAS studies should not be considered an appropriate substitute for confirmation in replication datasets.

### Association with Previously Reported Schizophrenia Loci

#### GWAS

To check for common findings between our study and previous schizophrenia GWAS studies, we first checked the six SNPs from O'Donovan *et al.* that remained significant after replication analyses [20]. None of the SNPs were directly genotyped on our platforms, however the three strongest associated SNPs, a well as two of the three others were represented by a proxy SNP with r^2^≥0.69. Since some of the Munich samples from this study were used in the replication for the O'Donovan *et al.* paper, we reported association statistics here for the Aberdeen samples only (Table S1). In the Aberdeen dataset, none of the SNPs showed a significant association with schizophrenia.

We then went on to check the p values for the following SNPs, or their closest proxy within 100 kb that was genotyped in our schizophrenia samples: the top 63 SNPs shown to be associated with individual genotyping in Kirov *et al* [19], the top 25 SNPs from Sullivan *et al* [14] and all individually genotyped SNPs that had a p<0.05 in combined males and females from Shifman *et al* [18]. These particular SNPs were chosen because only these p values were made publically available in the papers. Of these 116 SNPs, 8 were not represented in our dataset. Of the other 108, 6 were associated at p<0.05, and the lowest p value was 0.002 for rs11595716, a proxy for rs17746501, associated in Shifman *et al*. at p = 0.007 [18](see Table S2 for all data). This SNP is not located in a gene, nor is it strongly associated with any other SNP in or close to a gene. The other associated SNPs had p values of 0.011 (proxy for rs151222), 0.015 (proxy for rs234993), 0.020 (proxy for rs208799), 0.044 (rs4761874) and 0.048 (proxy for rs297257). Since rs151222 and rs234993 have an r^2^ of 1 with one another, these results are no more significant than what we would expect to see by chance when examining 116 SNPs.

Additionally, we examined rs7341475, a SNP proposed in this same study as a female-specific risk factor, in just the females from our study (n = 275 cases and 361 controls) and found no association (p = 0.24). Finally, a recent whole genome association study of schizophrenia [17] found that one polymorphism, rs4129148, achieved genome wide significance and a second associated common polymorphism, rs28414810, was in one of the nearest genes [17], both located in the pseudoautosomal region of chromosomes X and Y. As in the original paper, we tested the SNPs separately in males and females, but could find no evidence of association in males or females of either cohort using either an additive or a recessive model.

#### Candidate Genes

It has often been argued that associations in candidate genes with strong *a priori* hypotheses of disease involvement (due to previous association or due to biological plausibility) should be treated with more weight in a genome-wide association study than SNPs in other genes or in non-genic regions. We have therefore also reassessed a set of previously reported schizophrenia candidate genes with a less stringent correction for multiple testing [30]. For each gene we first checked whether a previously-associated candidate SNP itself (or a suitable proxy) showed any association in our cohort and secondly whether any SNP in the gene showed association in our cohort following correction for all the SNPs tested in the relevant gene (Table 2).

**Table 2:** Twenty-five schizophrenia candidate genes were checked for association with schizophrenia in a cohort of 879 cases and 864 controls.

*Gene*	*Previously associated SNPs present on Illumina chip*	*Previously associated SNP represented by an LD proxy on Illumina chip*	*Closest proxy (r^2^ in CEU)*	*SNP replication p*	*OR excluded*	*Lowest p*	*#SNPs tested in gene*	*Gene-wide correction*	*Set-wide correction*	*Genome-wide correction*
***AKT1***				NA	NA	0.416	4	1	1	1
***CAPON/NOS1AP***				NA	NA	**0.033**	45	1	1	1
***CHRNA7***				NA	NA	0.067	14	0.94	1	1
***COMT***	rs4680			0.94	1.05	0.090	12	1	1	1
***DAO***				NA	NA	0.288	9	1	1	1
***DAOA(G72)***	rs3916971 rs778293			0.09	1.23	0.085	10	0.85	1	1
***DISC1***	rs2295959 rs3738401			0.621	1.14	0.082	64	1	1	1
***DRD2***		rs6277 rs6275	rs754672 (0.81) rs2242592(1)	0.753	1.12	0.033	14	0.46	1	1
***DRD3***	rs6280			0.491	1.15	0.168	13	1	1	1
***DTNBP1***		rs760761	rs1474605 (1)	0.948	1.05	0.107	18	1	1	1
***ERBB4***	rs7598440 rs707284	rs839523 rs4673628	rs839517 (0.86) rs1851169(1)	0.313	1.18	**0.012**	181	1	1	1
***FEZ1***				NA	NA	**0.002**	13	**0.026**	1	1
***GAD1***				NA	NA	0.289	9	1	1	1
***GRIK4***				NA	NA	**0.028**	54	1	1	1
***GRM3***		rs1468412	rs2237562 (1)	0.495	1.15	0.075	27	1	1	1
***HTR2A***		rs6313	rs4941573 (1)	0.498	1.15	**0.042**	21	0.88	1	1
***MRDS1(OFCC1)***				NA	NA	**0.017**	36	0.61	1	1
***MUTED***				NA	NA	0.035	31	1	1	1
***NOTCH4***		rs175174	rs11089328(0.93)	0.872	1.09	**0.0017**	28	**0.048**	1	1
***NRG1***				NA	NA	**0.012**	157	1	1	1
***PPP3CC***				NA	NA	0.228	6	1	1	1
***PRODH2***				NA	NA	0.163	7	1	1	1
***RGS4***		rs951439	rs6678136 (1)	0.186	1.20	0.186	2	0.372	1	1
***TNF***				NA	NA	0.248	5	1	1	1
***ZDHHC8***		rs175174	rs11089328 (0.93)	0.872	1.09	0.794	2	1	1	1

Displayed are (1) gene symbol; (2) previously associated candidate SNPs in that gene, included in the Illumina 300K chip; (3) previously associated candidate SNPs in that gene, not included in the Illumina 300K chip but can be represented by a LD proxy in the chip; (4) the closest proxy SNP present on the Illumina 300K chip for column 3; (5) the lowest p value for SNP-specific (column 2 and 4) replication; (6) Maximum odds ratio (allelic OR under multiplicative genetic model) at the previously-implicated locus, based on observed p value at this SNP or a proxy. A real odds ratio of this size is expected to produce a p value as high as the one observed only 5% of the time, based on the power formulae of Chapman *et al* [59]; For detailed review on the previous reports of these candidate genes and SNPs see reference [30]; (7) lowest p value of all the HumanHap300 SNPs located in that gene (lowest p); (8) number of SNPs tested in each gene; (9) the lowest p value corrected for all the SNPs tested in that gene (gene-wide correction); (10) the lowest p value corrected for all the SNPs in the 25 candidate genes (set-wide correction); (11) the lowest p value after correction for all HumanHap300 SNPs tested (genome-wide correction).

None of the SNPs in these genes, however, shows a significant association with schizophrenia after correction for only those SNPs in the 25 previously-associated candidate genes (n = 782). If we restrict our correction to only the SNPs within single candidate genes, however, then two of the genes contain SNPs that remain following gene-wide correction: *FEZ1* (corrected for 13 SNPs) and *NOTCH4* (corrected for 28 SNPs). The most strongly associated SNP in *NOTCH4* (rs3134942, a synonymous coding SNP in very high LD with rs8192585, a nonsynonymous coding SNP) was actually the top hit in the Aberdeen cohort (p = 0.000016), but has not itself been implicated in other schizophrenia studies, and was not even marginally significant in the Munich dataset (p = 0.93).

Where we have been able to test a previously-associated SNP (or its proxy) in each relevant gene, we have also assessed the maximum effect size on schizophrenia risk that is consistent with our failure to see effects in this study. The maximum permitted allelic odds ratio is 1.23, and most have values close to 1.15, suggesting that these variants generally have little or no impact on disease susceptibility (see Table 2, ‘odds ratio excluded’ column). Although our data provide little support for the previously reported candidate genes, we recognize that if the real effect sizes are small, these genes may not stand up to correction for multiple testing (even when considering candidate genes as a set on their own). Additionally, it should be noted that for some reported loci, the Illumina SNP sets did not include the best-associated variants from previous studies (see Table 2, ‘original SNPs without proxy’ column).

Finally, we developed a framework to assess how informative our negative genome-wide association study is about the cumulative contribution of common SNPs to schizophrenia. To mimic our study design, we assumed that for a SNP to be detected it must both obtain p<0.0003 in the initial GWAS and obtain a joint p<1.6×10^−7^ when combined over the initial GWAS and the first subsequent replication stage (see Methods for details of power calculations). We find that it is possible for a single causal SNP, tagged at *r* ^2^ = 0.8 with a GWAS SNP and with MAF = 0.2, to be undetected with a 20% probability if the allelic odds ratio is less than 1.58. This means, not unexpectedly, that a single SNP with a relatively large odds ratio could easily have been missed in our study. But it is important to note that while one such SNP might easily be missed, many such SNPs could not be missed, and alone, such a SNP would contribute a locus specific to λ_S_ of only 1.04, which amounts to only a tiny fraction of the sibling relative risk in schizophrenia. To assess whether our negative data are consistent with the hypothesis of common variants explaining most of the sibling relative risk, we also investigated the relationship between the number of causal SNPs that might exist in the genome, given that all are undetected with 20% probability in our study, under a simple assumption that that all SNP effect sizes are equal and again assuming *r* ^2^ = 0.8 and MAF = 0.2 for each SNP. We find that for 2, 10 and 100 SNPs the limits on allelic odds ratios are 1.50, 1.38 and 1.27 respectively, while the combined contribution to total λ_S_ based on these SNPs (assuming they act independently on risk) are 1.07, 1.22 and 2.85 respectively. Taking these arguments to their logical conclusion, our data are also consistent with the possibility that 274 SNPs each with an OR = 1.22, *r* ^2^ = 0.8 and MAF = 0.2 lie undetected in the human genome, with 20% probability. The total contribution of these SNPs to λ_S_ would be 10, consistent with observed estimates [30]. This calculation must be viewed as hyper-conservative in terms of the contribution of common variation since it assumes that all contributed variants have an equal relative risk, while observations from other conditions make clear that effect sizes fall off after the first several that are discovered [31]. We also note that the pattern of reduction in λ_R_ moving from first to second to third degree relatives is more consistent with the presence of epistatic relationships among causal SNPs, rather than the independent model considered here [32].

### Copy Number Variation

For analysis of copy number variation, we used the three cohorts with genome-wide SNP genotype data, namely Aberdeen, Munich, and an American cohort that has not yet been studied (for copy number variation) in any previous publications. All samples that passed SNP-QC procedures (see Methods) were entered into the CNV analysis, whereupon further QC was performed to determine if accurate CNV calling could be expected (see Methods). In Aberdeen, 12 samples (10 cases and 2 controls) failed CNV QC, in Munich 39 samples (9 cases and 31 controls) and in the American 49 samples (29 cases and 14 controls) failed CNV QC. These samples were excluded from further analysis, leaving a final dataset of 422 cases and 381 controls from Munich, 441 cases and 439 controls from Aberdeen and 150 cases and 264 controls from the US (European origin), a total of 1,013 cases and 1,084 controls. We also examined both previously implicated regions, and regions newly implicated here in 60 African-American schizophrenia patients and 64 African American controls (after excluding 8 and 1 respectively for CNV QC failure).

#### Very large copy number variants

We first evaluated the frequencies of copy number variants above 500 kb in cases and controls to determine whether there are overall size thresholds above which copy number variants would appear to have a reasonably high prior likelihood of disease involvement based on rarity in controls (Table 3). We excluded all CNVs that had ≤20 contributing SNPs as these were all spanning centromeres - regions of very low SNP coverage, and likely to be false positives. For deletions, we found as the size of the CNV increased, there was a tendency towards increased frequency in cases compared to controls. This was not the case for duplications until the 2 Mb size threshold was reached. Above the 2 Mb size threshold (as depicted on these particular genome-wide platforms), we found that deletions were absent in controls. Based on their rarity in controls, we went on to examine in detail all events greater than 2 Mb (Table 4). In this category, we observed 17 events, comprising 8 deletions, all in cases, and 9 duplications, 6 in cases and 3 in controls (Table 4). Fisher's Exact test indicated that such very large events were significantly more prevalent in cases than controls (p = 0.006). However, when we separated these events into deletions and duplications we found that although deletions remained significantly more frequent in cases (p = 0.003), duplications did not (p = 0.33).

**Table 3:** Frequency of deletions in duplications in cases and controls from 500 kb to greater than 2 Mb.

	500 kb–1 Mb		1–1.5 Mb		1.5–2 Mb		2 Mb+	
	case	control	case	control	case	control	Case	control
**deletions**	0.013	0.013	0.009	0.004	0.002	0.0009	0.007	0
**duplications**	0.054	0.073	0.014	0.013	0.004	0.005	0.006	0.003

**Table 4:** All CNVs greater than 2 Mb.

Chr	start	end	SNPs	length	state	start SNP	end SNP	Status	Cohort	Notes
**1**	**144106312**	**146293282**	**260**	**2,186,971**	**del**	**rs10797649**	**rs2932454**	**case**	**Aberdeen**	**This deletion has been found in other schizophrenia samples [22],[24]. A 1.4 Mb deletion was also found in the US cohort at the same location (chr1:144838594–146293282).**
**1**	**144106312**	**146292286**	**259**	**2,185,975**	**del**	**rs10797649**	**rs2999617**	**case**	**Aberdeen**	
**8**	**15098795**	**18360001**	**536**	**3,261,207**	**del**	**rs12680924**	**rs7000897**	**case**	**Munich**	**Includes TUSC3, associated with mental retardation** [**47**] **, PCM1, which has been associated with schizophrenia ** [**48**] **, ASAH1, whose deficiency results in mental retardation ** [**49**] ** and NAT1 and NAT2 which have been implicated in schizophrenia.**
**16**	**15387380**	**18072544**	**491**	**2,685,165**	**del**	**rs251633**	**rs9284326**	**case**	**Aberdeen**	**Includes NDE1, which interacts with DISC1 to control neurodevelopmental processes ** **[25]**,**[34]**.
**22**	**16389909**	**19792353**	**668**	**3,402,445**	**del**	**rs174330**	**rs140392**	**case**	**Aberdeen**	**Region implicated in schizophrenia in multiple studies.**
**22**	**17257787**	**19792353**	**496**	**2,534,567**	**del**	**rs2543958**	**rs140392**	**case**	**Aberdeen**	
**22**	**17257787**	**19792353**	**496**	**2,534,567**	**del**	**rs2543958**	**rs140392**	**case**	**Aberdeen**	
**22**	**17257787**	**19792353**	**496**	**2,534,567**	**del**	**rs2543958**	**rs140392**	**case**	**Aberdeen**	
1	185089907	187703805	220	2,613,899	dup	rs2076075	rs3860306	case	Munich	PLA2G4A is the only gene affected by this event. This gene has previously been implicated in schizophrenia [63].
2	106190084	109173575*	455	2,983,492	dup	rs13006272	rs11893469	case	Aberdeen	Includes many genes, none previously implicated in schizophrenia.
4	96866611	99167973	156	2,301,363	dup	rs2865703	rs783919	case	Munich	PDHA2 in region, not good candidate gene.
4	179888441	182314469**	567	2,426,029	dup	rs436324	rs12503294	control	US	No genes in region
5	37621606	39730156	490	2,108,551	dup	rs665327	rs1499238	control	Aberdeen	Includes many genes, including good schizophrenia candidates, e.g. GDNF. However subject is unaffected.
13	79334893	81402686	381	2,067,794	dup	rs6563141	rs9531267	case	Aberdeen	Includes SPRY2, which shows decreased expression in brains of schizophrenia patients[64].
15	21240037	26208861	556	4,968,825	dup	rs4778531	rs1635168	case	Munich	Includes many genes, notably GABRA3 and GABRA5, overlaps with larger duplication found in US cohort.
15	21260328	30302218***	1600	9,041,890	dup	rs17118751	rs4779984	case	US	Overlaps both with duplication in Munich cohort and also region of previously reported schizophrenia-associated 1.4 Mb duplication encompassing APBA2 [40].
22	17343340	19792353	347	2,449,014	dup	rs2019061	rs140392	control	Munich	Same region deleted in cases. A 1.5 Mb duplication was also seen in a US case (chr22:17257787–18719310)

*: Inspection in BeadStudio indicates that this duplication is actually 4.06 Mb, extending from chr2: 106282170–110339905.

**: Inspection in BeadStudio indicates that this duplication is actually 2.91 Mb, extending to chr4: 182,802,687.

***: Inspection in BeadStudio indicates that this duplication is actually 9.41 Mb, extending to chr15: 30,544,756.

To investigate the role of deletions >2 Mb in healthy samples tested in an extensive battery of cognitive tests, we searched for such events in a set of 1,547 ethnically-mixed cognitively normal healthy controls. We were unable to find any sample with a deletion >2 Mb - the largest was 1.5 Mb. This indicates that deletions greater than 2 Mb are very rare (<0.04%) in the healthy, cognitively normal population, and suggests that when such very large deletions are found, they appear to have a high prior probability of being disease associated (although not necessarily predictive of schizophrenia [33]), even when occurring only in a single individual. Further analysis of much larger sample sizes of cognitively normal individuals will be required to validate this conclusion.

Of these very large deletions, four of eight were in the chromosome 22q11.2 region that has been previously associated with schizophrenia [34]. All four of these were in the Aberdeen cohort, giving a prevalence of approximately 1%. This is in accordance with the previously reported frequency of 0.75% (95%CI: 0.5%–1.2%) [35]. The Munich and US cohorts, however did not show any large deletions in this region, although one US case and one Munich control subject showed a duplication spanning the same region. Of the remaining four very large deletions, two, both in the Aberdeen cohort, spanned a 2.06 Mb region on 1q21.1 that has been previously reported in two larger studies using the same samples [22],[24], and has also been found in other populations [21],[22],[24]. We also found a 1.7 Mb deletion in the US cohort that overlapped with this (chr1:144,612,035–146,336,720), and ended at the same position.

**Figure 1:**
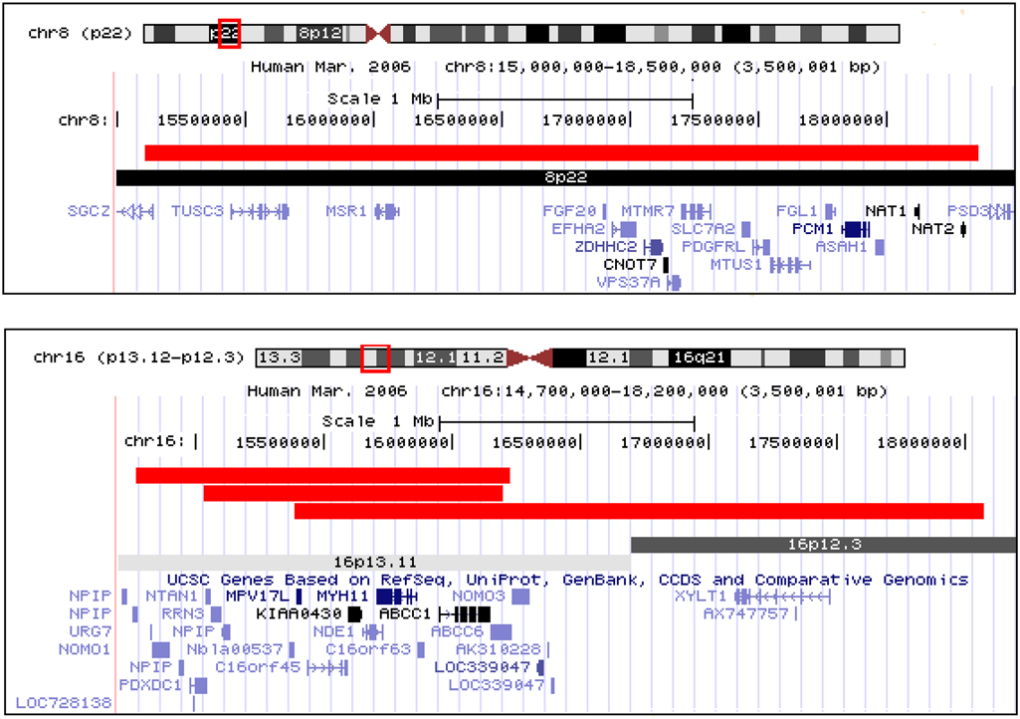
Novel >2 Mb deletions found in schizophrenia cases. Duplications and smaller deletions in region not shown. The chromosome 8 region is deleted in a single Munich patient. The chromosome 8 region is deleted in a patient from Aberdeen and has overlapping, smaller deletions in a patient from Munich and an African American patient. (Adapted from UCSC browser: http://genome.ucsc.edu).

Two of the 8 very large deletions are newly reported here as possible contributors to schizophrenia (Figure 1). One, also in the Aberdeen cohort, spanned a 2.69 Mb region on 16p13.11-p12.4. This region includes the gene *NDE1*, which binds to *DISC1* in brain developmental processes [24],[36]. *DISC1* is a gene that is disrupted in patients with schizophrenia and other severe neuropsychiatric disorders in one Scottish family [37]. Interestingly, in this region we also saw a 1.2 Mb deletion in a Munich sample, and a 1.5 Mb deletion in an African American patient (chr16:14,771,033–16,225,138). All three deletions included the region chr16:15387380–16198600 (and the genes *MPV17L*, *c16orf45*, *KIAA0430*, *NDE1*, *MYH11*, *KIAA0866*, *c16orf63*, *ABCC1* and *MRP6*/*AbCC6*) indicating that a large deletion of this region may be a recurrent schizophrenia risk factor. Further investigation of this region in 755 US epilepsy patients genotyped on the Illumina Human610-Quad BeadChip revealed a further 6 deletions >500 kb (Heinzen *et al.*, in preparation), suggesting that it is a risk factor for other neuropsychiatric conditions as well as schizophrenia. Collectively, these data strongly suggest that deletions in this regions contribute to schizophrenia and epilepsy, providing another example of a CNV influencing different neuropsychiatric conditions [38], and supporting the observation that deletions greater than 2 Mb are likely to be disease-associated.

The second newly-reported very large deletion was in the Munich cohort, spanning 3.25 Mb on 8p22 and includes a number of promising candidate genes (Table 4). Inspection of the region in other samples did not provide further support.

We also inspected each of the large duplications, although these were not unique to cases in our analyses. This suggests that large duplications can be compatible with normal cognitive function, making it more difficult to suggest causality to any of the large duplications in the cases. However, some of the duplications were of interest nevertheless. In the US cohort, we have one patient who has a 9.4 Mb duplication (reported as 9.04 by PennCNV, Table 4) on chromosome 15q11.2-13.3, which extends across the Prader-Willi/Angelman syndrome critical locus, a region known to contain many segmental duplications and inverted repeats [39] (the patient does not suffer from either Prader-Willi or Angelman syndrome). This overlaps two other large duplications - a 5 Mb duplication in a Munich case (Table 4), and a *de novo* 1.5 Mb duplication involving APBA2 previously reported in a schizophrenia patient [40]. Additionally, it overlaps with a previously reported schizophrenia-associated deletion event at chr15:28.72–30.30 [22],[24], and we also observed a 1.16 Mb deletion in this region in a US patient. This evidence confirms a role for recurrent mutation in this region in schizophrenia susceptibility and indicates that duplications as well as deletions of this region can lead to schizophrenia. Additionally, since no duplication greater than 3 Mb was found in any control subject, there is evidence that duplications of this size are detrimental in general.

#### Burden of rare CNVs greater than 100 kb

Next, we investigated general CNV load between cases and controls. It was recently shown that schizophrenia patients were more likely to have rare CNVs greater than 100 kb that disrupted genes (that is, began or ended within a gene) or that deleted or duplicated entire genes [21],[22], although this was not replicated in a Chinese population [41]. Following Walsh *et al.* [21], we selected all CNVs greater than 100 kb that had not been previously reported in the DGV and compared the frequencies of those that did and did not affect genes between cases and controls, separating cases and controls, and deletions and duplications. Since our CNVs are identified based on SNP data, we are not able to precisely determine where each event begins and ends. Therefore, we did not attempt to distinguish between “disrupted” (Walsh *et al.* define this as a gene that is interrupted by a CNV [21]) and “included” genes (genes completely encompassed by a CNV) - instead we counted all gene-including and apparently gene-disrupting CNVs together as “gene-affecting”.

First looking at the Aberdeen cohort, which has been included in a previous study replicating the Walsh *et al.* effect [22] we found that 91 of the 441 Aberdeen cases that passed QC (21%) contained one or more rare deletions >100 kb that affected a gene, compared to 66/439 controls (15%), and that 61/441 (14%) cases contained rare, greater than 100 kb, gene-affecting duplications, compared to 49/439 (11%) of controls. Fisher's exact test indicated that this was a significant excess of deletions in cases (p = 0.03), but only a trend for duplications (p = 0.26). In contrast, neither the Munich nor the US cohort (which have not been assessed in previous publications) showed an excess of deletions in cases (Table 5), although the Munich cohort had significantly more duplications in cases (p = 0.03) and US cohort showed a trend in the same direction for deletions (p = 0.08).

**Table 5:** Count of European-ancestry samples with one or more rare gene-affecting CNV that is greater than 100 kb and includes 20 or more SNPs.

	Aberdeen				Munich				Meltzer/memory			
	deletion		duplication		deletion		duplication		deletion		duplication	
	case	control	case	control	case	control	case	control	case	control	case	control
**Has 1 or more CNV that affects a gene**	91	66	61	49	30	29	61	36	13	11	20	43
**Total**	441	439	441	439	422	381	422	381	161	267	150	264
**Fisher's 1-tailed p value**	**0.034**		**0.112**		**0.788**		**0.030**		**0.079**		**0.897**	

It was also previously reported that there were no differences between cases and controls for rare CNVs greater than 100 kb that did not disrupt genes [21], however the International SNP Consortium found an excess of rare CNVs greater than 100 kb that do not disrupt genes in schizophrenia cases [22]. It should be noted that this dataset also included the Aberdeen samples. Our findings here, however, were similar to those of the gene-affecting CNVs: Aberdeen had significantly more rare deletions greater than 100 kb that did not affect a gene (two-tailed Fisher's p = 0.031), and no other comparisons were significantly different (Aberdeen duplications, Munich and US duplications and deletions). Overall, therefore, we cannot offer further support to the hypothesis that rare CNVs greater than 100 kb are present in excess in schizophrenia patients, although, as shown above, we report a trend for increased deletions greater than 1 Mb, and a significant excess of greater then 2 Mb deletions in cases.

The population used by Walsh *et al.* contained a large number of young-onset and childhood-onset schizophrenia patients [21], a population one might expect to be enriched for genetic rather than environmental contributors. Like the Walsh *et al.*, cohort, the Aberdeen schizophrenia cohort seems to contain an unusually large number of copy number variants both in comparison to the Aberdeen controls and in comparison to the other schizophrenia cohorts. However, the Aberdeen cohort was not enriched for young-onset patients, nor was it in any other obvious way different from the Munich patient cohort. The patients from both regions were selected using a consistent clinical protocol and the distribution of schizophrenia subtypes were similar. Further examination of population differences with those that do and do not carry an excess burden of large rare CNVs will be necessary to elucidate the differences between cohorts in this respect. Additionally, these differences seen in this study may in part depend on the type of platform used to detect the CNVs. The Aberdeen, Munich, and US cohorts were each genotyped using a different genotyping platform (Illumina HumanHap550, HumanHap300, Human-610 Quad respectively), using only partially overlapping SNP sets. This could influence the CNV calls, although it is difficult to see why the HumanHap550 platform would detect an excess of CNVs in comparison to the Human-610 Quad chip which is designed to have improved CNV detection.

#### Common CNVs in schizophrenia

We then tested whether any of the more common CNVs were significantly different in frequency between cases and controls, using Fisher's exact test. This has not been investigated in any of these samples previously. In this case common is a relative term rather than defined by a particular frequency cut-off, since a common CNV was defined as one that was present in three or more individuals in any particular cohort. We did not attempt to classify different CNVs as identical or different, as use of the different BeadChips can make the beginning and endpoints of the CNVs unclear. Additionally, the actual length of the CNV may be unimportant if it covers the same critical region as a shorter or longer CNV. We therefore performed this analysis by determining copy number for each SNP on the Beadchip, and performing Fisher's Exact test for each SNP included in a CNV. Low confidence CNVs, those in some telomeric and centromeric regions and those coding for immunoglobulin genes were removed (see Methods) before beginning the analysis, since these are particularly susceptible to false positive calls.

We first tested the Aberdeen, Munich and US samples separately, and also separately compared a) deletions, b) duplications, and c) genomic regions affected by both types of CNV. The Munich cohort had 1,299 SNPs affected by deletions (with frequency ranging from 0.4% to 15%), 1,042 SNPs affected by duplications (0.04%–9.3%) and 202 SNPs affected by both deletions and duplications (in different subjects; frequency 0.5%–9.4%). The equivalent values for Aberdeen were 3,879 deletions (0.34%–18.9%), 2,634 duplications (0.34%–17.5%) and 1,016 SNPs affected by both (0.45%–62%) and for the US cohort, 2,702 deletions (0.72%–46.7%), 3,159 duplications (0.72%–21%) and 1,399 SNPs affected by both (1.0%–58%). It should be noted that these CNVs have not been individually validated either by inspection in BeadStudio or by any experimental means, and many of these are likely to be false positives. The three cohorts differed both in the CNVs that most strongly associated and in the direction of the effects. There were no events that were significantly associated (p<0.05) in more than one cohort.

To test for effects of common CNVs that may not be well captured by PennCNV calling algorithms from Infinium HumanHap SNP data, we next examined a set of SNPs shown to tag copy number polymorphisms (CNPs), as defined in McCarroll *et al.* [42] and provided by Drs. McCarroll and Altshuler (personal communication). Of 285 total HapMap tagging SNPs, we found that 169 and 202 of them were either directly represented or had a proxy of r^2^≥0.8 on the HumanHap310 and Human610-quad BeadChips, respectively. We then specifically examined the association statistics for these CNP-tagging SNPs in Aberdeen and Munich (combined) and in the US cohort. None of the association p values stood up to correction for the total SNPs tested, neither did any CNP-tagging SNP associate at p<0.05 (uncorrected) in both populations. We can therefore provide no evidence for the role of common CNVs in schizophrenia. However, it should be noted that many common CNVs cannot be detected using the HumanHap300 and 550 genotyping platforms [42], so it is not possible for us to conclusively rule out effects of common CNVs on schizophrenia.

We then searched for very highly penetrant schizophrenia loci that were present in one or more cohorts. We did this by performing Fisher's exact test on all three cohorts combined (see Methods) and searching for significantly associated events that were present only in cases, and that occurred in cases from at least two cohorts. According to these criteria, we found 4 associated regions of deletion and 3 associated regions of duplications. After excluding regions that were common in the DGV, we were left with just one region of interest: chr11:112772031–112778135, with six deletions in the Aberdeen cohort (one 40 kb from 112,744,722–112,784,640, two 23 kb from 112761718–112784640, two 7 kb from 112772032–112779220 and one 6.1 kb from 112772032–112778135) and one in the US cohort (9.3 kb, from 112775371–112784640). These positions were confirmed by visual inspection in BeadStudio. This region included the 3′ end of the ANKK1 gene and was immediately downstream of the DRD2 gene, a target of all antipsychotic drugs. It is possible that this region could contain regulatory regions relevant to the dopamine D2 receptor function, and the functional effects of this CNV, as well as its presence in other cohorts, should be further investigated. It should be noted, however, that 1/1,547 cognitively normal subject in the extra control cohort also carried this deletion, so if it is a risk factor it has incomplete penetrance.

#### Inspection of previously reported schizophrenia-associated CNVs

Using comparative genomic hybridization, Kirov *et al.* reported 13 possible schizophrenia associated CNVs [40]. One of these, a 1.4 Mb duplication including *APBA2* and other genes was also found in our cohort and has already been discussed. The other CNV they designated as most likely to be pathogenic was a 250 kb deletion of 2p16.3 that included the 5′ end of *NRXN1*, a gene also implicated in two other recent studies [43],[44]. In our cohort we had three large deletions that encompassed the 3′ end of *NRXN1* (200 kb in US, 260 kb and 420 kb in Munich), providing further evidence for this region in schizophrenia pathology. (It should be noted, however, that we also observed *NRXN1* deletions in the cognitive normal extra control cohort, so all deletions in this gene should not be assumed to be pathogenic.) They also reported a 240 kb duplication encompassing the *EFCAB2* and *KIF26B* genes, and we found a 575 kb duplication of this region in one African-American patient. Thirdly, we also found two large duplications encompassing a reported 640 kb non-genic duplicated region on 4q35.2: 1.26 Mb in Munich and 1.28 Mb in Aberdeen. In summary, of 13 reported schizophrenia associated CNVs, we were able to find supporting evidence for 4 in our cohorts, including both of their highest-confidence regions [40]. Another study reported deletions of *CNTNAP2* in schizophrenic patients [45], but we did not find any large events in this gene, and smaller deletions were present in both cases and controls. We next searched our cohorts for any of the 21 novel autosomic schizophrenia events reported by Walsh *et al.* [21]. We have already reported 2 Aberdeen and 1 US subject with the 1.4 Mb deletion on chromosome 1, which is now a known recurrent schizophrenia risk factor [22],[24]. Of the remaining 20, we found only 1 event that reoccurred in our samples: 2 Aberdeen cases had a 664 kb duplication at chr2:48625109–49290093. In a fourth study, Stone *et al.* (also using the Aberdeen cohort, genotyped with a different platform) reported the presence of duplications in the *NOTCH1* and *PAK7* genes in cases only [22]. We did not find any duplications in *NOTCH1*, however we found one deletion in an Aberdeen sample and three deletions in cases from the US cohort, and none in controls. We also found one duplication in *PAK7* in a US case, and none in controls. Finally, a small study using genome-wide SNP discovery on 54 patients with deficit schizophrenia found and validated four rare schizophrenia-associated CNVs [44], two deletion affecting *NRXN1* (see above), and *ASTN2*, and two duplications affecting 4 and 7 genes respectively, at 2p16.3 and 5p15.2. In *ASTN2* we saw three very small deletions in Munich cases and two small deletions in a Munich and an Aberdeen control. In the 2p16.3 region (chr2:859,616–1,826,716), we found a 441 kb deletion in a Munich case, and two large duplications in a control (250 kb and 237 kb), from 836164–1086540 and 1589418–1826014. This indicates that rare duplications in this area are not highly-penetrant schizophrenia risk factors. At 5p15.2 (chr5:10,270,604–11,200,814) we observed only 4 very small deletions, all in Munich controls.

#### Analysis of pathways affected by gene-disrupting CNVs in cases and controls

After finding an excess of rare gene-disrupting CNVs greater than 100 kb in their schizophrenia patients, Walsh *et al.* [21] went on to investigate the genes using the Ingenuity Pathway Analysis (IPA) classification system to check in cases for an excess of disrupted genes from any particular functionally defined molecular pathway. They found a significant over-representation of genes in pathways important for brain development. We performed the same analysis on the genes that were disrupted by rare CNVs greater than 100 kb in our cohorts (Table S3). We did not find a strong overlap with the pathways enriched in Walsh *et al.* [21], nor did we find common pathways disrupted in cases across the three cohorts we examined.

## Discussion

While our genome-scan identified no definitive associations between SNPs and schizophrenia risk, SNPs in the *ADAMTSL3* gene were the most strongly associated. One of the *ADAMTSL3* SNPs is clearly functional and influences the proportion of two alternative transcript species. Nevertheless, study of additional cohorts strongly suggested that *ADAMTLS3* is not related to schizophrenia risk and that functional evidence should not be used to strengthen claims for modestly associated variants.

We also failed to replicate any of the SNPs previously identified in either genome-wide association studies or candidate gene studies as schizophrenia risk factors. Notably, not only did none of these show genome-wide significance, none showed significant evidence even if we corrected only for the 782 SNPs from previously associated candidate genes.

We have calculated here that by using genome-wide genotyping in the discovery cohorts and then typing the top 100 SNPs in the first replication cohort, we have 80% power to detect an allele with MAF≥0.1 at an odds ratio of 1.8 or larger. This is one of the largest whole genome association studies reported for schizophrenia and is similar in size or larger than studies that have successfully identified risk factors for common diseases [46],[47]. While our sample size is not large enough to identify risk factors of small effect in a genome-wide context, we have introduced an approach for assessing whether our negative results are consistent with a model in which common SNPs explain most of the heritability of schizophrenia. While this analysis shows that we cannot rule out such a possibility, it would require a large number of SNPs and an implausible genetic model.

There are a number of possible explanations for the lack of compelling genetic associations in schizophrenia GWAS to date. Firstly, it has been postulated that schizophrenia is not an homogenous condition, but is in fact a group of several different genetically heterogeneous syndromes that are classed together due to overlap of particular diagnostic symptoms [48]. This hypothesis may be tested in the near future with the publication of collaborative datasets combining several thousand cases and controls, which will presumably include reasonable sample sizes of each subtype. However, in order to distinguish between the presence of reasonable effect sizes in subgroups of the patients and very small effect sizes common to all, it will be necessary to examine more detailed information about the patients including symptoms, putative disease subtypes, and perhaps measures of cognition and other endophenotypes.

Similarly, large datasets would also be necessary to investigate the possibility that common variants exert their effects only through interaction with other genetic variants, a plausible hypothesis that it has not yet been possible to investigate in any powerful way. Alternatively, genetic risk factors may interact so strongly with the environment that their marginal effects over all environments are too low to detect in a sample of this size. Further investigation of this will require much larger, longitudinal studies of patients and controls.

The final possibility is that much of schizophrenia risk is due to rare, moderate-to-high penetrance variants whose population frequencies reside somewhere below the threshold of detection of genome-wide screens. Due to the complex patterns of schizophrenia heredity, and the relative lack of families with Mendelian schizophrenia syndromes, this cannot account fully for schizophrenia susceptibility. On balance, however, the data presented here are most consistent with this interpretation. First, we find no evidence of association for common SNPs, but clear evidence that a fraction of cases are due to very rare, very highly penetrant structural variants. One interpretation of this pattern is that selection for reliable cognitive function has been sufficiently strong to keep the genome free of common variants that predispose to schizophrenia, and that it is only rare deleterious variants that influence risk [49]. This model for schizophrenia genetics presents clear challenges to the hope that genetics will rapidly reveal new therapeutic opportunities or partition patients up into a small number of clinically manageable subgroups.

More encouragingly, however, despite the rarity of these events, we nonetheless replicated several specific regions from previous studies that were not known to be recurrent schizophrenia-associated CNVs, including those affecting *APBA2* and the surrounding region [40]. Additionally, we have implicated new regions, including a large deletion at 16p13.11-p12.4 that may be an important risk factor for other neuropsychiatric conditions. This region also intimates at the possibility that while patients may have different genetic contributors, some of the different events may point towards the same pathway, given that the 16p region includes a gene known to encode a binding partner of DISC1, a gene with confirmed involvement in schizophrenia. These observations suggest that a full catalogue of rare determinants of schizophrenia could identify a number of specific genomic regions or events that unite a fraction of patients as having the same or similar underlying causes. These subgroups of patients can be further investigated to see if the genetic contributors can elucidate the molecular mechanisms underlying particular symptoms or drug-response phenotypes.

Finally, even if most of the schizophrenia risk is due to rare relatively highly penetrant causes, it seems unlikely this would all be structural. It is likely that rare single site changes disrupting gene function must contribute as well, and will likely only be determined through full genome resequencing, which must be considered a goal for future schizophrenia genomic research.

## Methods

### Ethics Statement

All cases and controls gave informed consent. The study was approved by both local and multiregional academic ethical committees.

### Discovery Cohort

The SNP discovery cohort consisted of two distinct sub-cohorts. The first cohort comprised 439 schizophrenia patients (age 39.2±10.4 yr, range 19–70) and 418 healthy controls (age 48.8±14.7 yr, range 22–75), all self-identifying as of German or central European ancestry and collected in Munich. The second cohort comprised 461 schizophrenia patients and 459 controls, all self-identifying as of Scottish or north European ancestry, collected in Aberdeen, Scotland. Critically, patients for the two cohorts were selected using a consistent clinical protocol. To be enrolled as a case, participants must have had both a DSM-IV and an ICD-10 diagnosis of schizophrenia [50]. In the Munich and Aberdeen cohorts respectively, subtypes were observed in the following proportions: paranoid 77.6% and 86.2%, disorganized 15.6% and 7.5%, catatonic 2.2% and 2.1% and undifferentiated 4.6% and 4.2%. Detailed medical and psychiatric histories were collected, including a clinical interview using the Structured Clinical Interview for DSM-IV (SCID), to evaluate lifetime Axis I and II diagnoses. Cohen's Kappa (Cohen, 1960 [51]) of 0.80 indicated good inter-rater reliability. Exclusion criteria included a history of head injury or neurological diseases. All case participants were outpatients or stable in-patients. Further details of the Munich cohort and protocol are available in Van den Oord *et al.* (2006) [52]. All cases and controls gave informed consent. The study was approved by both local and multiregional academic ethical committees.

Healthy volunteers were randomly selected from the general population both for the Munich and Aberdeen cohorts (ascertained by mail for Munich, and by general practitioners for Aberdeen). In the Aberdeen study volunteers were screened for absence of psychiatric disorders and only those with no major psychiatric episodes or major mental illness in a first degree relative were included in the study. In the Munich cohort several screenings were conducted before the volunteers were enrolled in the study in order to exclude subjects with central neurological diseases and psychotic disorders or subjects who had first-degree relatives with psychotic disorders. First, subjects who responded were screened by phone for the absence of neuropsychiatric disorders. Second, detailed medical and psychiatric histories were assessed for the volunteers and their first-degree relatives using systematic forms. Third, if no exclusion criteria were fulfilled, they were invited to a comprehensive interview including the SCID [52] to validate the absence of psychotic disorders. Finally, a neurological examination was conducted to exclude subjects with current CNS impairment. In the case that the volunteers were older than 60 years, the Mini Mental Status Test [53] was performed to exclude subjects with possible cognitive impairment. The enrolment procedure was similar for the Aberdeen controls, although a formal SCID was not undertaken.

### First Replication Cohort

The first replication cohort comprised 298 schizophrenia patients (age 37.3±11.8 yr, range 18–66) and 713 healthy controls (age 45.5±16.1 yr, range 19–72). The recruitment protocol is identical to that used for the Munich discovery sample. Schizophrenia subtypes were observed in the following proportions: paranoid 75.5%, disorganized 16.1%, catatonic 4.7% and undifferentiated 3.7%.

### Second Replication Cohort

The sample comprised a total of 918 subjects of whom 394 (mean age±SD 43.5±12.8 years, range 19–80) had a DSM-IV-TR [50] diagnosis of schizophrenia and 524 (mean age±SD 47.3±29.7 years, range 19–87) were healthy controls. Patients and controls were of Caucasian ancestry for at least two generations, lived in northern Italy, were unrelated to other participants, and fulfilled predefined group-specific inclusion and exclusion criteria. The different subtypes of schizophrenia were observed as follows: paranoid 61.9%, undifferentiated 17.0%, residual 10.4%, disorganized 9.4%, catatonic 1.3%. The patients were enrolled from those voluntarily admitted to the Brescia IRCCS Fatebenefratelli. The inclusion criteria were a DSM-IV-TR diagnosis of schizophrenia [54] and a level of understanding and attention judged sufficient to give true informed consent; a lifetime comorbidity with other DSM-IV-TR Axis I disorders was an exclusion criterion. All participants underwent detailed clinical interviews, implemented, when required, by DSM-IV-TR adjusted versions of the Structural Clinical Interview for DSM-IV Axis I Disorders. Moreover, to attribute the schizophrenia subtype, a checklist of the symptoms dominating the clinical picture at the screening visit and in the previous 4 weeks was used.

All patients and controls enrolled in the study provided a written informed consent approved by local Ethical Committee (CEIOC, Brescia, Italy). A concise, but unequivocal explanation about the aims of the study was included on the written consent form.

The healthy unrelated participants were recruited through different sources (randomly selected among university, consenting doctors, nurses, employees and attendants of Brescia IRCCS Fatebenefratelli and elderly association). All participants underwent a psychiatric interview to exclude Axis I disorders and Axis I diagnosis of first-degree relatives. Absence of relevant neurological diseases was mandatory for the inclusion in the study. The Mini Mental Status Test 29 was performed to subjects older than 65 years, to exclude possible cognitive impairment.

### Third Replication Cohort

For the third replication, we used information provided by Dr. Hreinn Stefánsson by personal communication, with reference to the deCODE samples used in previous publications, e.g. [24].

### US Cohort Used for CNV Analysis and as Fourth SNP Replication Cohort

The US patients were part of an NIMH-funded Clinical Research Center at Case Western Reserve University and prospective clinical trials at Vanderbilt University. Information about recruitment and assessment has been previously reported [55],[56]. The healthy controls were recruited as part of the Genetics of Memory/ Genetics of Epilepsy studied at Duke. All subjects were cognitively normal and free of neuropsychiatric disorders.

### Extra Control Cohort (used to Search for Deletions >2 Mb)

The extra 1,547 controls were also part of the Genetics of Memory/ Genetics of Epilepsy studied at Duke and genotyped in the same facility using the Illumina Infinium HumanHap 550K, and subject to identical quality control procedures. They comprised healthy controls who performed normally in a series of cognitive tests (age range = 18–85, mean = 25.5, median = 22). The majority were of European origin but also included were approximately 10% each of African-American, East Asian (mostly Chinese) and South Asian (mostly Indian) as well as 5% Hispanic.

### Genotyping and Quality Control

The Munich cohort was genotyped using the Illumina HumanHap300 chip with a total of 317,503 SNPs and the Aberdeen cohort was genotyped using the Illumina HumanHap550 chip with a total of 555,352 SNPs. We carried out a series of quality control (QC) checks and tests of cryptic relatedness, ultimately excluding a total of 15 and 28 participants in Munich and Aberdeen respectively (Text S1). We also employed a “one percent rule” that discarded from analysis any SNP that had more than 1% of samples that could not be reliably scored, to reduce the scope for spurious association. After employing this rule the average success rate of genotyping was 98.4% and the concordance rate for duplicate genotyping was 99.997%. The US cohort was genotyped using the Human-610 Quad Beadchip at the Institute for Genome Sciences and Policy Genotyping Core, and the same quality control procedures were applied as those used for the discovery cohorts.

### Association Analyses and Correction for Population Structure

Our core association analyses to identify schizophrenia risk factors focused on single-marker tests of the 312,565 QC-passed SNPs that were genotyped in both cohorts. To control for the possibility of spurious associations resulting from population stratification we used the EIGENSTRAT approach of Price *et al* [57]. This method derives the principal components of the correlations among gene variants and corrects for those correlations in the association tests. In principle, therefore the principal components in the analyses should reflect population ancestry. We have noticed however that some of the leading axes appear to depend on other sources of correlation, such as sets of variants near one another that show extended association. We have documented the potential for inversions to create this effect and it may be created by other causes of extended linkage disequilibrium as well (Text S1). For this reason we inspected the SNP ‘loadings’ for each of the leading axes to determine if they depended on many or relatively few SNPs, as would be expected if the given axis reflected population ancestry or a more localized linkage disequilibrium effect respectively. This analysis identified several axes clearly due to inversions and suggested that four axes should be retained for ancestry adjustment (Text S1). We therefore assessed significance using four principal components emerging from the EIGENSTRAT analyses as covariates in a logistic regression model which also incorporated sex as a covariate and combined samples from Munich and Aberdeen (a division which clearly drove the first EIGENSTRAT axis).

### Bayesian Analysis of Posterior Odds of Association

Following Wakefield [29], we found the estimated log-odds for association, 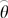, under a multiplicative genetic model for rs2135551, together with its estimated variance *V*, from standard logistic regression of each dataset. Given a prior odds of *PO* for the association being true, and a prior distribution of ∼N(μ,W) for θ under the hypothesis of true association, we found the posterior odds having observed new data at each stage as 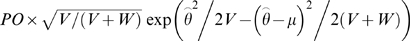, and updated the posterior distribution of θ under the hypothesis of true association as 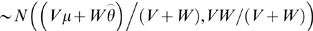. We then entered these posteriors as priors into the analysis of the next set of data. To start, we set *PO* = 1/100000 following the Wellcome Trust Case Control Consortium [58] (i.e., assuming a million independent regions of the genome and 10 detectible causal loci for schizophrenia), and following Wakefield, 2007 [29] we set μ = 0 and W = (log(1.5)/1.96)^2^ (i.e., assuming that 95% of all casual effects fall between 2/3 and 3/2 per allele under a multiplicative genetic model).

### Alternative Splicing

Alternative transcripts were identified searching ExonHit Therapeutics SpliceArray portal (http://portal.splicearray.com) and blasting exon-intron boundary sequences against human cDNA libraries. For semi-quantitative evaluation of transcript ratio differences, primers flanking the common 5′ splice donor site in exon 29 (forward primer: 5′-TTGGGCCCTCCTGTGATA-3′, location shown in Figure S1A) and the alternative 3′ splice acceptor site in exon 30 (reverse primer: 5′-TGGCAGCACCTTTGTTTGTA-3′, location shown in Figure S1A) were used to simultaneously amplify all four transcript forms (Figure S1A). The fragments were separated on a 3.5% NuSieve agarose gel and direct sequencing was used to confirm expected transcript forms. Taqman-based real time PCR was used to quantitatively determine ratios of alternative transcripts in human brain tissue. Assays were custom designed through Applied Biosystems by targeting unique exon-exon boundaries (for primer and probe sequences see Text S1). β-actin mRNA expression level was quantified using a commercially available Taqman assay (Applied Biosystems). Fluorescence outputs were quantified in real time using a 7900HT Fast Real Time PCR System and the data were analyzed using SDS software v.2.2.2 (Applied Biosystems). One way analysis of variance was used to determine the correlation of alternative transcript abundance with the rs950169 and rs2135551 genotypes in human brain tissue. Statistical analyses were performed both separately in control and Alzheimer's disease prefrontal cortex samples, and as a combined subject analysis. A genomic DNA fragment of 4028 bp from the *ADAMTSL3* gene that included exons 29 and 30 with flanking intron sequences was PCR-amplified from a reference genomic DNA using the following primers: gggaattcAAGGGCAGATACCCCAAAGT and taggatccCGCTTGCTCTTCCAACTACC. Subsequently, the PCR fragment was subcloned into pSPL3 (GibcoBRL) as a minigene. The minor allele of rs950169 was generated in the minigene by mutagenesis (QuikChange Mutagenesis kit, Stratagene) and the sequences were confirmed by DNA sequencing. The minigenes were transfected into HEK293 cells using Lipofectamine2000 (Invitrogen). After the 48 h transfection, RNA was extracted using RNeasy kit (Qiagen) and converted into cDNA using High-Capacity cDNA Archive Kit (Applied Biosystems). Alternative splicing of exon 29 and exon 30 was detected by Taqman assays and agarose gel.

### Power Calculations

Following Chapman *et al.* [59], we assumed that the test statistic from a case-control trend test of association follows a non-central chi-square distribution with 1 d.f. and non-centrality parameter *η* = (*n*−1)*r* ^2^ *H*, where *n* is the sample size, *r* ^2^ is the LD between the causal SNP and it's tag SNP on the GWAS genotyping panel, and *H* is the proportion of variation explained by the SNP if it were typed directly. In a case-control setting, 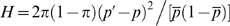, where *Π* is the proportion of cases in the total sample, *p* is the frequency of causal alleles in controls and in the general population (assuming a rare disease), *p*′ is the frequency of causal alleles in cases (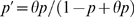 where *θ* is the allelic relative risk or odds ratio assuming a rare disease), and 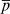 is the causal allele frequency in the study as a whole. We simulated sets of 100,000 *X*_1_ values from a Normal distribution with mean = √*η*_1_ and variance = 1, where *η*_1_ is the presumed non-centrality parameter from the GWAS study (*n* = 1734, *Π* = 0.506), and an additional set of 100,000 *X*_2_ values from a Normal distribution with mean = √*η*_2_ and variance = 1, where *η*_2_ is the presumed non-centrality parameter from the first replication study (*n* = 1011, *Π* = 0.295). To score a “hit”, we required both that *X*_1_ exceeded the upper critical value for a two-tailed test at α = 0.0003 (to mimic being passed to the 1^st^ replication stage), and that both *X*_1_ and *X*_2_ had the same sign and had a joint *P*<1.6×10^−7^ when combined using Stouffer's weighted-Z method [60] (to mimic achieving a Bonferroni-corrected genome-wide significance level after both stages). Power was defined as the number of hits divided by 10,000. Solutions to *θ* based on fixed values of the other parameters were found by an iterative root-finding procedure (function “uniroot” in the R statistical package, http://www.r-project.org/). The Total Lambda-s expected based on *k* independent SNPs each with a given OR and MAF was found using equations in Camp *et al.* [61].

### Generation of CNV Calls and QC

All subjects that passed SNP QC procedures were entered into the CNV analysis. This comprised 892 samples from Aberdeen (441 controls, 451 cases), 842 samples from Munich (412 controls, 430 cases) and 443 samples from the US (267 controls, 176 cases). The CNV calls were generated using the PennCNV software (version 2008jun26 version [62]) using the Log R ratio (LRR) and B allele frequency (BAF) measures automatically computed from the signal intensity files by BeadStudio, and the standard hg18 “all” PennCNV hidden Markov model (hmm) and population frequency of B allele (pfb) files for the 317 and 550 BeadChips. For the samples genotyped on the 610-Quad BeadChips, we used the hh550_610.hg18 pfb and gc model files separately provided by Dr. Kai Wang to ensure inclusion of all CNV-specific markers. Because many of the samples had below optimal genomic wave QC values, for Aberdeen and Munich we implemented the gc model wave adjustment procedure. We used the PennCNV checks to exclude samples that failed quality control. These included samples that had a LRR standard deviation >0.28, BAF median>0.55 or <0.45, BAF drift >0.002 or WF>0.04 or <−0.04. For the US cohort we found an excess of CNVs in samples with LRR_SD values between 0.25 and 0.28, so the LRR_SD cut-off was reduced to 0.25 for both cases and controls from the US. All samples that failed QC after the wave adjustment procedure were removed. Due to the complications of hemizygosity in males and X-chromosome inactivation in females, all analyses were restricted to autosomes. Additionally, to ensure that we were working with high-confidence CNVs, we excluded any CNV for which the difference of the log likelihood of the most likely copy number state and the less likely copy number state was less than 10 (generated using the -conf function in PennCNV). Finally, some centromeric and telomeric regions are not well mapped, and this can potentially result in CNV-calling errors in these regions (Dr. Kai Wang, personal communication). Also, genomic regions coding for immunoglobulin genes have previously been shown to be potential sites of false-positive PennCNV calls [62]. Our own research has shown that calls in both of these types of region differed significantly depending on the sample type used for DNA extraction (significant difference p<10^−10^ for deletion and/or duplication frequencies between samples genotyped on DNA extracted from blood or saliva, data not shown). We therefore excluded any CNV that overlapped any of the following regions by 50% or more of its length: chr2: 87.0–92.0, chr14: 18–23.6 Mb, chr14: 104.5–106.5 Mb, chr15: 17.0–21.0, chr16: 31.8–36.0 Mb, chr22: 20.5–21.8 Mb (immunoglobin regions); chr1:0–4 Mb, 240–247 Mb; chr2: 87.0–92.0 Mb; chr4: 0–1.43 Mb, 48.75–49 Mb, 190.7–191.3 Mb; chr7:0–200 kb, 56.5–62.5 Mb; chr8: 39–45 Mb, 145–146.3 Mb; chr9: 44.5–70.1 Mb; 138–140.2 Mb; chr10: 38.5–42 Mb, 134–135.4 Mb; chr11: 0–1.8 Mb; chr14: 18–23.6 Mb, 104.5–106.5 Mb; chr15: 17.0–21.100–100.3 Mb; chr16: 0–2.1 Mb, 31.8–36.0 Mb, 86.6–88.9 Mb; chr17:0–1 Mb, 76.5–78.8 Mb; chr18: 14–16 Mb, 75.5–76 Mb; chr19: 0–2.1 Mb, 25.7–28.3 Mb, 61.5–62.5 Mb; chr20: 25.7–28.3 Mb, 61.5–62.5 Mb; chr21:9.7–14.3 Mb; chr22:14.4–14.7 Mb, 20.5–21.8 Mb (centromeric and telomeric regions, some overlapping immunoglobin regions as above). We also removed CNVs that spanned centromeres by searching for those larger than 1 Mb with fewer than 50 SNPs and checking their genomic locations.

### Analysis of Rare CNVs Greater than 100 kb

Following Walsh *et al.*, we defined rare copy number variations as those with at least 100 kb in size, at least 20 SNPs and not previously described the Database of Genomic Variants (DGV, http://projects.tcag.ca/variation/; dgv18v6). Any previously described event that had at least a 60% overlap with a newly discovered event was considered ‘not rare’ and excluded from further evaluation (for details see [21]). We then looked to see if there was an increase in particular types of rare CNVs between cases and controls using a 2-tailed Fisher's Exact test to compare number of cases versus number of controls with and without the event.

### Common CNV Analysis

In order to implement a genome-wide screen for the effect of common CNVs on schizophrenia predisposition, the number of deletions and duplications affecting each SNP was counted up and compared between cases and controls using Fisher's exact test. For each population, separate analyses were done for deletions, duplications and loci affected by both deletions and duplications. To enter the deletion analysis, a SNP had to be deleted in 3 or more samples and duplicated in fewer than 2 samples, for the duplication analysis a SNP had to be duplicated in 3 or more samples and deleted in fewer than 2 samples and the third analysis included all SNPs that are deleted in 2 or more samples and duplicated in 2 or more samples. Events that only occurred in one or two individuals were not analyzed. For the screen for schizophrenia-specific recurring events, we performed the same statistical test, and again stipulated that the event must occur in at least three individuals, but this time we did not filter out sites that were affected by duplications from the deletion analysis nor those affected by deletions from the duplication analysis, in order to maximize the search space for each test.

### Pathway Analysis

Firstly, we screened the genes that are affected by the rare CNVs greater than 100 kb (described above). To do this we mapped all genomic coordinates for SNPs used in defining CNVs into the most updated human genome variation build (Ensembl variation build 50_36l, dbSNP build 129). We then aligned the genomic coordinates of the rare CNVs with the most updated human genome build (Ensembl core build 50_36l, human genome build 36). Any protein-coding gene that was either broken by or fully included in a rare CNV was considered “affected”. We detected the following gene counts that were affected by deletions in cases and controls respectively in the different populations: Aberdeen: 407, 210; Munich: 109,55; US: 70,159;and for duplications, Aberdeen: 294,180; Munich: 217,127; US: 34,17. We then used Ingenuity Pathway Analysis (IPA, see [21]) to perform a pathway enrichment (over-presenting) analysis separately for the four groups of genes we detected. The statistical significance was evaluated using Fisher's exact test. To avoid false positives, we further stipulated that at least two genes in a pathway must be disrupted for that pathway to be considered enriched, in addition to the P values from Fisher's exact test.

### Further information

#### Data availability

The results of this genome-scan are released temporarily for this submission at URL:

http://people.genome.duke.edu/˜dg48/samba/prjx89z/DUKE_IGSP_PG2_SCHIZO.zip

These results can be directly loaded and annotated using the custom-designed WGA (whole genome annotator) software, WGAViewer:

http://www.genome.duke.edu/centers/pg2/downloads/wgaviewer.php

These results will be publicly released through Mart for IGSP Data from Association Studies (MIDAS) once published. MIDAS can be directly accessed using the WGAViewer software.

## Supporting Information

Figure S1 Schematic of exons 29–30 of the *ADAMTSL3* gene and evidence of the presence of four alternative transcripts. A. Exons 29 and 30 are depicted as blue boxes and the PLAC domain coding part of gene is striped. The alternative splice donor site (ASD) and alternative splice acceptor site (ASA) are indicated by the black lines, while the locations of *ADAMTSL3* rs950169 and rs2135551 are indicated by the red stars. We also resequenced the indicated region and found no new candidate causal polymorphisms. We did however find a new rare variant (IVS29+5G>A, indicated by a red star) in the reference splice donor site (in the plus five position) which influences the usage of the reference donor site (data not shown). This variant showed a frequency of less than one percent in our cohort and was therefore too rare to properly assess any possible contribution of this new splicing variant to schizophrenia risk, although a protective trend was observed (data not shown). The black arrows represent location of primers used for semi-quantitative evaluation of alternative transcript ratios. Schema is not to scale. B. The amino acid sequence of *ADAMTSL3* protein PLAC domain and predicted effect of alternative splicing of exons 29 and 30. PLAC domain characteristic cysteines are highlighted. C. Evidence of the presence of four alternative *ADAMTSL3* transcripts in human brain tissue samples with different *ADAMTSL3* rs950169 and IVS29+5G>A genotypes. Lane 1: 100 bp ladder; Lanes 2 and 3: rs950169: CC, IVS29+5G>A: GG; Lanes 4 and 6: rs950169: CT, IVS29+5G>A: GG; Lanes 5 and 7: rs950169: TT, IVS29+5G>A: GG; Lanes 8 and 9: rs950169: CT, IVS29+5G>A: GA.
(1.85 MB TIF)

Figure S2 Evidence of the genetic control of *ADAMTSL3* exon 30 alternative splicing in human brain tissue. Correlation of the *ADAMTSL3* rs950169 genotype with the relative abundance of transcripts containing shorter exon 30 due to usage of alternative splice acceptor site (RSD-ASA) and full reference exon 30 (RSD-RSA) in the prefrontal cortex of control (•) and Alzheimer's disease brain tissue (◊). Bars indicate means of combined controls and Alzheimer's disease patients transcript abundance values. p<0.0001, ANOVA, combined controls and Alzheimer's disease patients p<0.0001, ANOVA, separate analyses of controls and Alzheimer's disease patients.
(0.48 MB TIF)

Figure S3 Evidence of the genetic control of *ADAMTSL3* exon 30 alternative splicing in human brain tissue. Correlation of the *ADAMTSL3* rs950169 genotype with the relative abundance of transcripts in the prefrontal cortex of control (•) and Alzheimer's disease brain tissue (◊). Bars indicate the means of combined controls and Alzheimer's disease patients transcript abundance values. A. Effect on alternative splice acceptor site in relation to alternative splice donor site. B. Effect on alternative splice donor site in relation to alternative splice acceptor site. C. No effect on alternative splice donor site in relation to reference splice acceptor site.
(0.79 MB TIF)

Figure S4 The expression of *ADAMTSL3* in the mouse forebrain as depicted in the Allen Brain Atlas. Highlighted is high expression in the pyramidal cell layer of the hippocampal formation (including CA1 and CA3 regions). We used information provided by the Allen Mouse Brain Atlas (http://www.brain-map.org) to determine the likelihood that a gene classified as showing expression in the brain at some point in development would show the same pattern of expression in the mouse brain as found in *ADAMTSL3*. Only 1.4% of all such genes (893/20598) showed clustered expression in the hippocampus.
(7.32 MB TIF)

Table S1 Association results in this dataset presented for the Aberdeen cohort for loci implicated in O'Donovan et al. [1].
(0.04 MB DOC)

Table S2 Association results in this dataset for SNPs previously implicated in schizophrenia GWAS studies.
(0.15 MB DOC)

Table S3 Pathway analysis for genes disrupted by large rare copy number variations in schizophrenia patients and controls.
(0.12 MB DOC)

Text S1 Supplementary methods and results.
(0.09 MB PDF)
