# Correction: A Genome-Wide Investigation of SNPs and CNVs in Schizophrenia

**DOI:** 10.1371/annotation/e0196ebb-de40-453f-8f8c-791b126618da

**Published:** 2009-03-24

**Authors:** Anna C. Need, Dongliang Ge, Michael E. Weale, Jessica Maia, Sheng Feng, Erin L. Heinzen, Kevin V. Shianna, Woohyun Yoon, Dalia Kasperavičiūtė, Massimo Gennarelli, Warren J. Strittmatter, Cristian Bonvicini, Giuseppe Rossi, Karu Jayathilake, Philip A. Cola, Joseph P. McEvoy, Richard S. E. Keefe, Elizabeth M. C. Fisher, Pamela L. St. Jean, Ina Giegling, Annette M. Hartmann, Hans-Jürgen Möller, Andreas Ruppert, Gillian Fraser, Caroline Crombie, Lefkos T. Middleton, David St. Clair, Allen D. Roses, Pierandrea Muglia, Clyde Francks, Dan Rujescu, Herbert Y. Meltzer, David B. Goldstein

The authors declare the following competing interest, which should have been declared at the time of publication. Pamela L. St. Jean, Pierandrea Muglia, and Clyde Francks are full-time employees of GlaxoSmithKline, a pharmaceutical company that is developing treatments for schizophrenia and that has filed patent applications for SNPs related to schizophrenia (United States Patent Applications 20080176239 and 20080176240, and International Application No.: PCT/EP2008/050477).

