# Supplementary figures and images for: A Genome-Wide Investigation of SNPs and CNVs in Schizophrenia
Source: PLoS Genet. 2009 Feb 6;5(2):e1000373. doi: 10.1371/journal.pgen.1000373 (PMC2631150; doi:10.1371/journal.pgen.1000373)

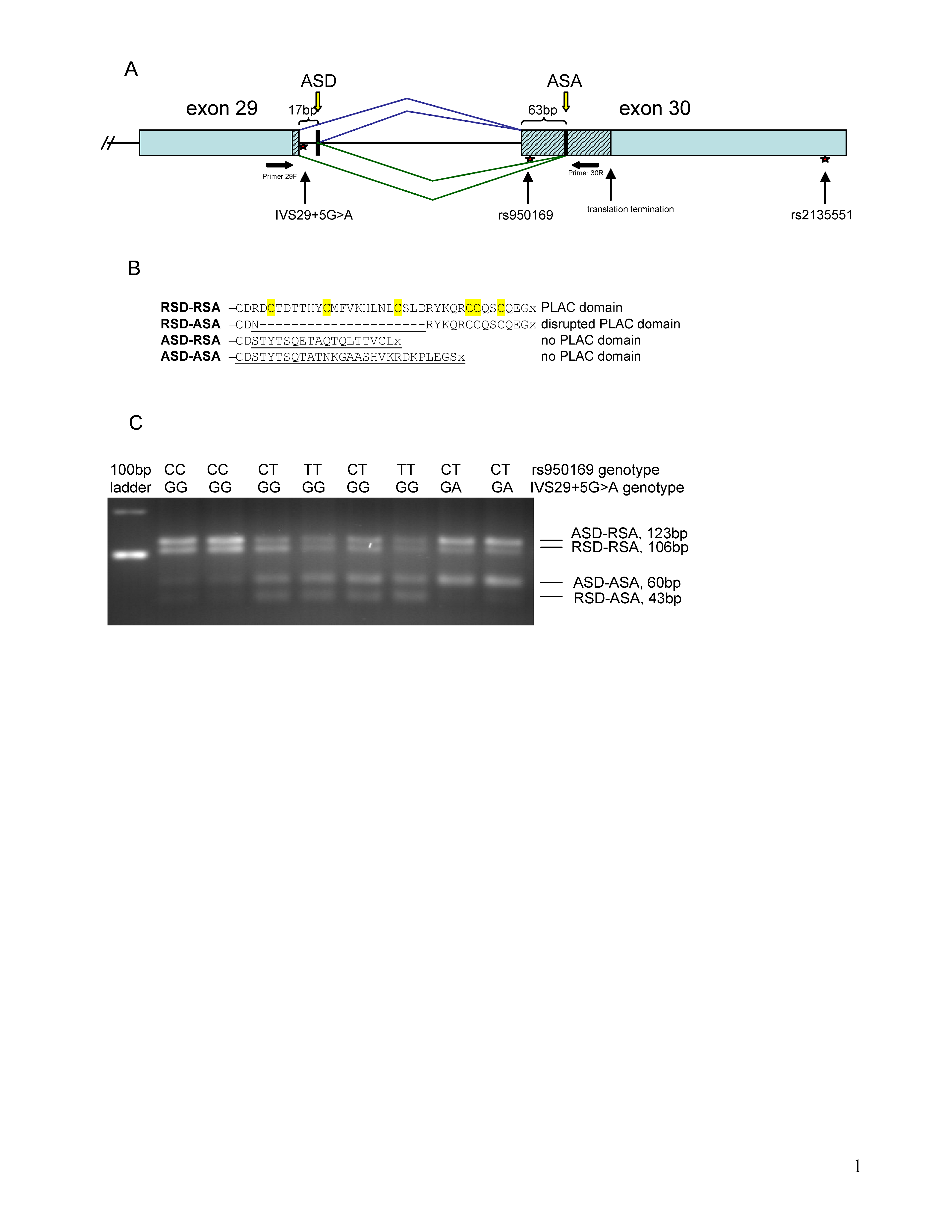

Supplement: Figure S1 — Schematic of exons 29–30 of the ADAMTSL3 gene and evidence of the presence of four alternative transcripts. A. Exons 29 and 30 are depicted as blue boxes and the PLAC domain coding part of gene is striped. The alternative splice donor site (ASD) and alternative splice acceptor site (ASA) are indicated by the black lines, while the locations of ADAMTSL3 rs950169 and rs2135551 are indicated by the red stars. We also resequenced the indicated region and found no new candidate causal polymorphisms. We did however find a new rare variant (IVS29+5G>A, indicated by a red star) in the reference splice donor site (in the plus five position) which influences the usage of the reference donor site (data not shown). This variant showed a frequency of less than one percent in our cohort and was therefore too rare to properly assess any possible contribution of this new splicing variant to schizophrenia risk, although a protective trend was observed (data not shown). The black arrows represent location of primers used for semi-quantitative evaluation of alternative transcript ratios. Schema is not to scale. B. The amino acid sequence of ADAMTSL3 protein PLAC domain and predicted effect of alternative splicing of exons 29 and 30. PLAC domain characteristic cysteines are highlighted. C. Evidence of the presence of four alternative ADAMTSL3 transcripts in human brain tissue samples with different ADAMTSL3 rs950169 and IVS29+5G>A genotypes. Lane 1: 100 bp ladder; Lanes 2 and 3: rs950169: CC, IVS29+5G>A: GG; Lanes 4 and 6: rs950169: CT, IVS29+5G>A: GG; Lanes 5 and 7: rs950169: TT, IVS29+5G>A: GG; Lanes 8 and 9: rs950169: CT, IVS29+5G>A: GA. (1.85 MB TIF) [file pgen.1000373.s001.tif]

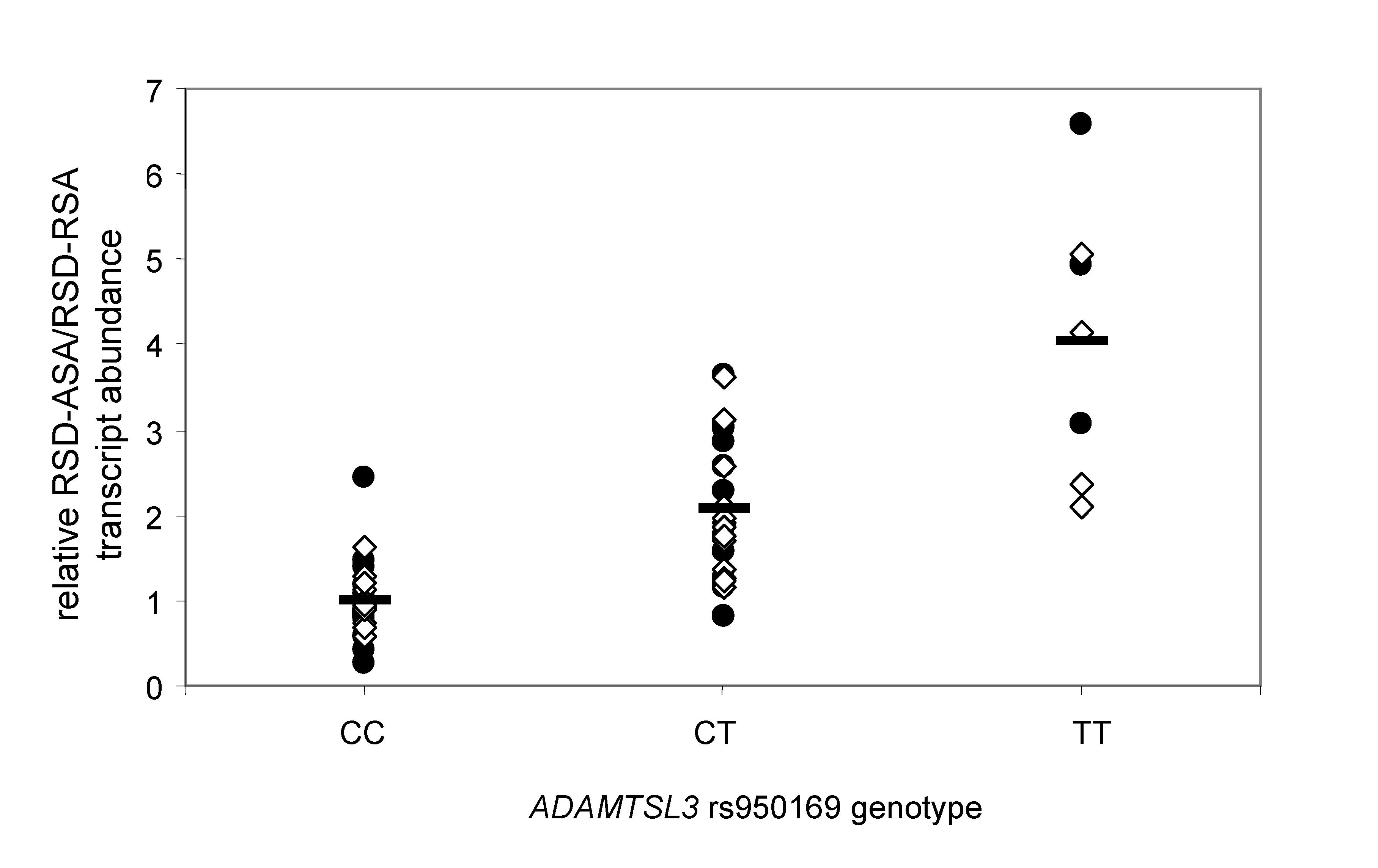

Supplement: Figure S2 — Evidence of the genetic control of ADAMTSL3 exon 30 alternative splicing in human brain tissue. Correlation of the ADAMTSL3 rs950169 genotype with the relative abundance of transcripts containing shorter exon 30 due to usage of alternative splice acceptor site (RSD-ASA) and full reference exon 30 (RSD-RSA) in the prefrontal cortex of control (•) and Alzheimer's disease brain tissue (◊). Bars indicate means of combined controls and Alzheimer's disease patients transcript abundance values. p<0.0001, ANOVA, combined controls and Alzheimer's disease patients p<0.0001, ANOVA, separate analyses of controls and Alzheimer's disease patients. (0.48 MB TIF) [file pgen.1000373.s002.tif]

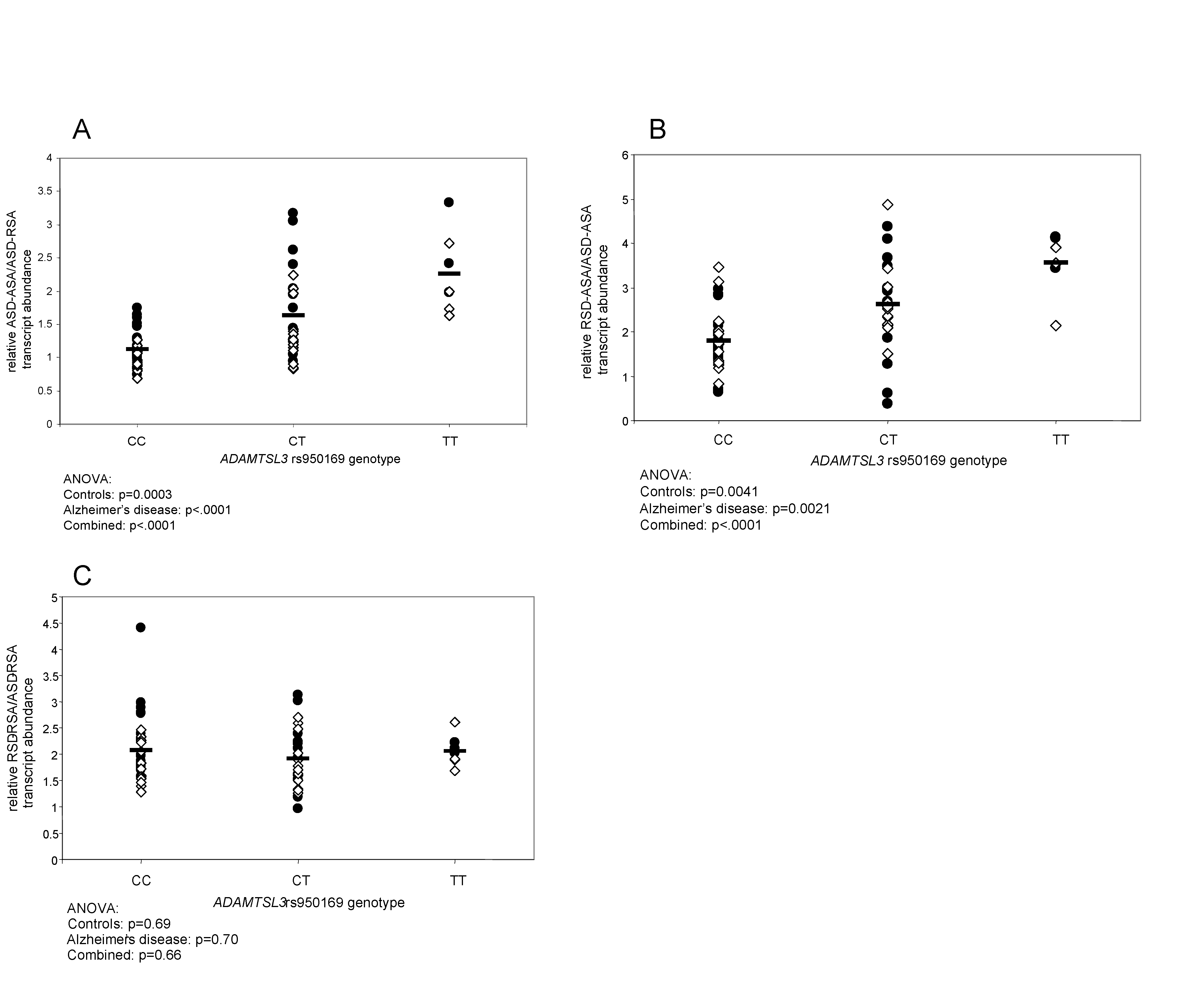

Supplement: Figure S3 — Evidence of the genetic control of ADAMTSL3 exon 30 alternative splicing in human brain tissue. Correlation of the ADAMTSL3 rs950169 genotype with the relative abundance of transcripts in the prefrontal cortex of control (•) and Alzheimer's disease brain tissue (◊). Bars indicate the means of combined controls and Alzheimer's disease patients transcript abundance values. A. Effect on alternative splice acceptor site in relation to alternative splice donor site. B. Effect on alternative splice donor site in relation to alternative splice acceptor site. C. No effect on alternative splice donor site in relation to reference splice acceptor site. (0.79 MB TIF) [file pgen.1000373.s003.tif]

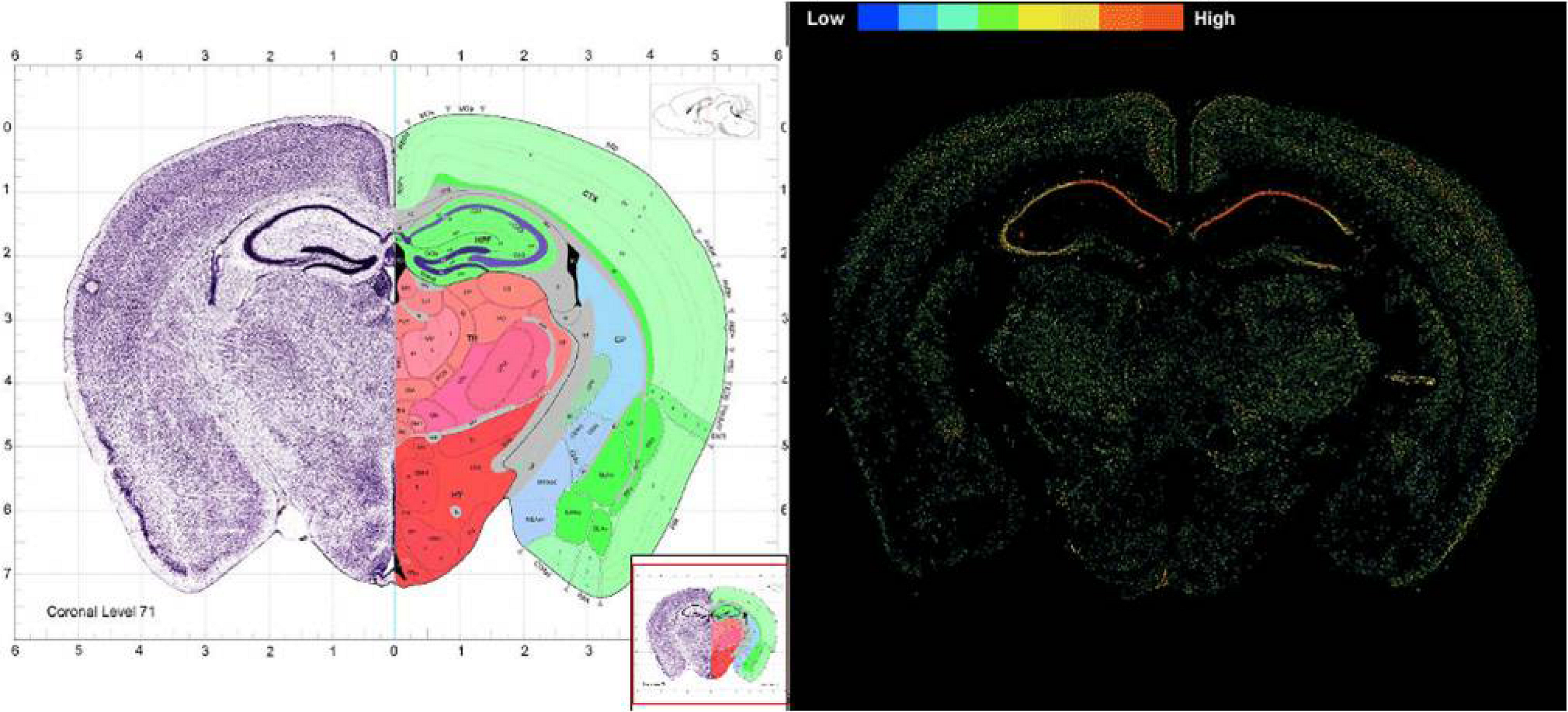

Supplement: Figure S4 — The expression of ADAMTSL3 in the mouse forebrain as depicted in the Allen Brain Atlas. Highlighted is high expression in the pyramidal cell layer of the hippocampal formation (including CA1 and CA3 regions). We used information provided by the Allen Mouse Brain Atlas (http://www.brain-map.org) to determine the likelihood that a gene classified as showing expression in the brain at some point in development would show the same pattern of expression in the mouse brain as found in ADAMTSL3. Only 1.4% of all such genes (893/20598) showed clustered expression in the hippocampus. (7.32 MB TIF) [file pgen.1000373.s004.tif]
