# Supplementary material for: A Genome-Wide Investigation of SNPs and CNVs in Schizophrenia
Source: PLoS Genet. 2009 Feb 6;5(2):e1000373. doi: 10.1371/journal.pgen.1000373 (PMC2631150; doi:10.1371/journal.pgen.1000373)
Supplement: Table S1 — Association results in this dataset presented for the Aberdeen cohort for loci implicated in O'Donovan et al. [1]. (0.04 MB DOC) [file pgen.1000373.s005.doc]

**Table S1. Association results in this dataset presented for the Aberdeen cohort for loci implicated in O’Donovan et al. [1].** Note that rs9922369 did not have a good proxy in our genotyping platform.

| **SNP** | **on chip?** | **Proxy** | **r2** | **SNP p value reported for partially overlapping Munich sample in original report** | **p value Aberdeen** |
| --- | --- | --- | --- | --- | --- |
| rs1344706 | no | rs1366840 | 0.69 | 0.021 | 0.4113 |
| rs1602565 | no | rs2933158 | 0.75 | 0.013 | 0.8928 |
| rs3016384 | no | rs7119286 | 0.69 | 0.141 | 0.816 |
| rs6490121 | no | rs2293054 | 0.75 | 0.283 | 0.3004 |
| rs7192086 | no | rs7189560 | 0.96 | 0.016 | 0.1452 |
| rs9922369 | no | rs2111119 | ***0.25*** | 0.265 | 0.3155 |

**Reference**

1. O'Donovan MC, Craddock N, Norton N, Williams H, Peirce T, et al. (2008) Identification of loci associated with schizophrenia by genome-wide association and follow-up. Nat Genet advanced online publication.
