# Supplementary material for: A Genome-Wide Investigation of SNPs and CNVs in Schizophrenia
Source: PLoS Genet. 2009 Feb 6;5(2):e1000373. doi: 10.1371/journal.pgen.1000373 (PMC2631150; doi:10.1371/journal.pgen.1000373)
Supplement: Table S2 — Association results in this dataset for SNPs previously implicated in schizophrenia GWAS studies. (0.15 MB DOC) [file pgen.1000373.s006.doc]

**Table S2. Association results in this dataset for SNPs previously implicated in schizophrenia GWAS studies.**

| Study | Associated SNP | Genotyped here? | Proxy | r2 between associated SNP and proxy | p value |
| --- | --- | --- | --- | --- | --- |
| Kirov et al., 2008 | rs4761874 | yes |  |  | 0.044 |
| Kirov et al., 2008 | rs12034664 | no | rs11247868 | 0.626 | 0.075 |
| Kirov et al., 2008 | rs13084692 | no | rs996966 | 0.231 | 0.094 |
| Kirov et al., 2008 | rs2029099 | yes |  |  | 0.095 |
| Kirov et al., 2008 | rs2659504 | no | rs2850982 | 1 | 0.118 |
| Kirov et al., 2008 | rs2395174 | yes |  |  | 0.126 |
| Kirov et al., 2008 | rs17231292 | no | rs2973343 | 0.266 | 0.128 |
| Kirov et al., 2008 | rs546464 | no | rs589248 | 0.591 | 0.131 |
| Kirov et al., 2008 | rs2007451 | no | rs874752 | 0.892 | 0.136 |
| Kirov et al., 2008 | rs10484735 | yes |  |  | 0.145 |
| Kirov et al., 2008 | rs4833722 | no | rs1588722 | 0.957 | 0.154 |
| Kirov et al., 2008 | rs6078931 | no | rs6078938 | 0.935 | 0.157 |
| Kirov et al., 2008 | rs5752019 | no | rs2179876 | 1 | 0.177 |
| Kirov et al., 2008 | rs6926332 | yes |  |  | 0.194 |
| Kirov et al., 2008 | rs11064768 | no | rs7977458 | 0.705 | 0.223 |
| Kirov et al., 2008 | rs2372441 | yes |  |  | 0.259 |
| Kirov et al., 2008 | rs11203820 | no | rs2055092 | 0.624 | 0.282 |
| Kirov et al., 2008 | rs2985662 | yes |  |  | 0.302 |
| Kirov et al., 2008 | rs1463535 | yes |  |  | 0.302 |
| Kirov et al., 2008 | rs9883916 | yes |  |  | 0.335 |
| Kirov et al., 2008 | rs7172362 | yes |  |  | 0.341 |
| Kirov et al., 2008 | rs768214 | yes |  |  | 0.363 |
| Kirov et al., 2008 | rs12455939 | no | rs517725 | 0.924 | 0.376 |
| Kirov et al., 2008 | rs4767235 | no | rs714253 | 1 | 0.401 |
| Kirov et al., 2008 | rs6950779 | yes |  |  | 0.412 |
| Kirov et al., 2008 | rs16934812 | no | rs61803 | 0.744 | 0.413 |
| Kirov et al., 2008 | rs1996794 | yes |  |  | 0.419 |
| Kirov et al., 2008 | rs1188568 | no | rs1188543 | 0.532 | 0.439 |
| Kirov et al., 2008 | rs10518356 | yes |  |  | 0.458 |
| Kirov et al., 2008 | rs7930681 | no | rs2341434 | 0.915 | 0.532 |
| Kirov et al., 2008 | rs176512 | yes |  |  | 0.536 |
| Kirov et al., 2008 | rs9548798 | yes |  |  | 0.553 |
| Kirov et al., 2008 | rs3759700 | no | rs10136093 | 0.879 | 0.553 |
| Kirov et al., 2008 | rs6027861 | yes |  |  | 0.554 |
| Kirov et al., 2008 | rs17035181 | no | rs983473 | 0.492 | 0.558 |
| Kirov et al., 2008 | rs1478684 | yes |  |  | 0.575 |
| Kirov et al., 2008 | rs9345837 | yes |  |  | 0.577 |
| Kirov et al., 2008 | rs946442 | yes |  |  | 0.595 |
| Kirov et al., 2008 | rs893703 | no | rs12493507 | 1 | 0.609 |
| Kirov et al., 2008 | rs11782269 | yes |  |  | 0.668 |
| Kirov et al., 2008 | rs6657332 | yes |  |  | 0.705 |
| Kirov et al., 2008 | rs6926853 | no | rs1325046 | 1 | 0.717 |
| Kirov et al., 2008 | rs512089 | yes |  |  | 0.729 |
| Kirov et al., 2008 | rs1510881 | yes |  |  | 0.731 |
| Kirov et al., 2008 | rs11144978 | yes |  |  | 0.738 |
| Kirov et al., 2008 | rs930767 | yes |  |  | 0.746 |
| Kirov et al., 2008 | rs980616 | yes |  |  | 0.764 |
| Kirov et al., 2008 | rs10461669 | no | rs2329656 | 0.965 | 0.813 |
| Kirov et al., 2008 | rs9457631 | yes |  |  | 0.818 |
| Kirov et al., 2008 | rs13406291 | yes |  |  | 0.851 |
| Kirov et al., 2008 | rs8045220 | no | rs4106010 | 0.773 | 0.859 |
| Kirov et al., 2008 | rs10509722 | yes |  |  | 0.896 |
| Kirov et al., 2008 | rs532210 | no | rs1580785 | 0.707 | 0.899 |
| Kirov et al., 2008 | rs320203 | no | rs320280 | 0.892 | 0.923 |
| Kirov et al., 2008 | rs424970 | yes |  |  | 0.943 |
| Kirov et al., 2008 | rs7122479 | no | rs10501622 | 0.383 | 0.95 |
| Kirov et al., 2008 | rs629310 | no | rs311346 | 0.961 | 0.972 |
| Kirov et al., 2008 | rs6965651 | no | rs6966462 | 0.588 | 0.978 |
| Kirov et al., 2008 | rs2288039 | yes |  |  | 0.993 |
| Kirov et al., 2008 | rs12455836 | no | no |  | none |
| Kirov et al., 2008 | rs16874040 | no | no |  | none |
| Kirov et al., 2008 | rs12224013 | no | no | 0.263 | none |
| Kirov et al., 2008 | rs17692695 | no | no |  | none |
| Shifman et al., 2008 | rs17746501 | no | rs11595716 | 0.887 | 0.002 |
| Shifman et al., 2008 | rs208799 | no | rs415256 | 0.592 | 0.02 |
| Shifman et al., 2008 | rs6908109 | no | rs997765 | 1 | 0.055 |
| Shifman et al., 2008 | rs7341475 | yes |  |  | 0.056 |
| Shifman et al., 2008 | rs3733975 | no | rs4867894 | 0.955 | 0.098 |
| Shifman et al., 2008 | rs6836567 | no | rs873090 | 0.93 | 0.125 |
| Shifman et al., 2008 | rs10903118 | no | rs10903122 | 0.931 | 0.128 |
| Shifman et al., 2008 | rs1177844 | no | rs1176486 | 0.816 | 0.167 |
| Shifman et al., 2008 | rs2073746 | yes |  |  | 0.191 |
| Shifman et al., 2008 | rs12521041 | yes |  |  | 0.219 |
| Shifman et al., 2008 | rs13192234 | no | rs11964846 | 1 | 0.285 |
| Shifman et al., 2008 | rs676269 | no | rs544606 | 0.96 | 0.313 |
| Shifman et al., 2008 | rs2938774 | no | rs4616917 | 0.429 | 0.365 |
| Shifman et al., 2008 | rs10419036 | no | rs971366 | 1 | 0.371 |
| Shifman et al., 2008 | rs2059087 | no | rs1422363 | 1 | 0.378 |
| Shifman et al., 2008 | rs1160752 | yes |  |  | 0.413 |
| Shifman et al., 2008 | rs839511 | no | rs839525 | 0.898 | 0.475 |
| Shifman et al., 2008 | rs4442642 | yes |  |  | 0.507 |
| Shifman et al., 2008 | rs10199440 | no | rs1837449 | 1 | 0.544 |
| Shifman et al., 2008 | rs4582985 | no | rs6589832 | 0.481 | 0.654 |
| Shifman et al., 2008 | rs3956319 | no | rs3019159 | 0.748 | 0.673 |
| Shifman et al., 2008 | rs2670115 | no | rs816828 | 0.934 | 0.679 |
| Shifman et al., 2008 | rs592523 | no | rs665677 | 0.805 | 0.827 |
| Shifman et al., 2008 | rs6598198 | no | rs7136649 | 0.938 | 0.915 |
| Shifman et al., 2008 | rs4085876 | no | rs3778652 | 0.963 | 0.934 |
| Shifman et al., 2008 | rs6586003 | no | rs1870167 | 0.943 | 0.939 |
| Shifman et al., 2008 | rs17666976 | no | rs323078 | 0.932 | 0.982 |
| Shifman et al., 2008 | rs7791506 | no | no |  | none |
| Sullivan et al., 2008 | rs151222 | no | rs11074471 | 0.391 | 0.011 |
| Sullivan et al., 2008 | rs234993 | no | rs7498905 | 0.391 | 0.015 |
| Sullivan et al., 2008 | rs297257 | no | rs297226 | 1 | 0.048 |
| Sullivan et al., 2008 | rs16977195 | no | rs2346713 | 0.274 | 0.272 |
| Sullivan et al., 2008 | rs9512730 | no | rs1535638 | 0.914 | 0.332 |
| Sullivan et al., 2008 | rs9309325 | yes |  |  | 0.336 |
| Sullivan et al., 2008 | rs17070578 | no | rs1361540 | 0.5 | 0.425 |
| Sullivan et al., 2008 | rs10521865 | no | rs7883888 | 0.234 | 0.474 |
| Sullivan et al., 2008 | rs1495716 | yes |  |  | 0.488 |
| Sullivan et al., 2008 | rs9295938 | no | rs3871466 | 0.796 | 0.505 |
| Sullivan et al., 2008 | rs1380272 | no | rs10516398 | 1 | 0.602 |
| Sullivan et al., 2008 | rs2824301 | no | rs208894 | 0.966 | 0.652 |
| Sullivan et al., 2008 | rs2159767 | no | rs2536576 | 1 | 0.661 |
| Sullivan et al., 2008 | rs2536589 | no | rs2536576 | 1 | 0.661 |
| Sullivan et al., 2008 | rs952515 | no | rs2536576 | 1 | 0.661 |
| Sullivan et al., 2008 | rs17095545 | no | rs10132666 | 1 | 0.735 |
| Sullivan et al., 2008 | rs7144633 | no | rs10132666 | 0.663 | 0.735 |
| Sullivan et al., 2008 | rs17455133 | no | rs11659290 | 1 | 0.784 |
| Sullivan et al., 2008 | rs9400690 | no | rs717389 | 0.917 | 0.803 |
| Sullivan et al., 2008 | rs942348 | no | rs7991497 | 0.87 | 0.825 |
| Sullivan et al., 2008 | rs10911902 | no | rs10798056 | 0.948 | 0.918 |
| Sullivan et al., 2008 | rs1569351 | no | rs727476 | 0.967 | 0.931 |
| Sullivan et al., 2008 | rs16917897 | no | no |  | none |
| Sullivan et al., 2008 | rs4568102 | no | no |  | none |
| Sullivan et al., 2008 | rs4846033 | no | no | 1 | none |
