# Supplementary material for: A Genome-Wide Investigation of SNPs and CNVs in Schizophrenia
Source: PLoS Genet. 2009 Feb 6;5(2):e1000373. doi: 10.1371/journal.pgen.1000373 (PMC2631150; doi:10.1371/journal.pgen.1000373)
Supplement: Table S3 — Pathway analysis for genes disrupted by large rare copy number variations in schizophrenia patients and controls. (0.12 MB DOC) [file pgen.1000373.s007.doc]

**Table S3. Pathway analysis for genes disrupted by large rare copy number variations in schizophrenia patients and controls.**

| **Pathway** | **Walsh et al.** | **dels in Aberdeen cases** | **dels in Aberdeen controls** | **dups in Aberdeen cases** | **dups in Aberdeen controls** | **dels in Munich cases** | **dels in Munich controls** | **dups in Munich cases** | **dups in Munich controls** | **dels in US cases** | **dels in US controls** | **dups in US cases** |
| --- | --- | --- | --- | --- | --- | --- | --- | --- | --- | --- | --- | --- |
| **Nitric oxide signaling** | 0.0002 |  |  |  |  |  |  |  |  |  |  |  |
| **Synaptic long-term potentiation** | 0.0005 |  |  |  |  |  |  |  |  |  |  |  |
| **Glutamate receptor signaling** | 0.003 |  |  |  |  |  |  |  |  |  | 0.041 |  |
| **ERK/MAPK signaling** | 0.004 |  |  |  |  |  |  |  |  |  |  |  |
| **PTEN signaling** | 0.007 |  |  |  |  |  |  |  |  |  |  |  |
| **IGF-1 signaling** | 0.008 |  |  |  |  |  |  |  |  |  |  |  |
| **Neuregulin signaling** | 0.008 |  |  |  |  |  |  |  |  |  |  |  |
| **Axonal guidance signaling** | 0.015 |  |  |  |  |  |  |  |  |  |  |  |
| **Synaptic long-term depression** | **0.017** |  |  |  |  |  |  | **0.010** |  |  |  |  |
| **G protein–coupled receptor signaling** | 0.034 |  |  |  |  |  |  |  |  |  |  |  |
| **Integrin signaling** | **0.036** | **0.021** |  | **0.039** |  |  |  |  |  |  |  |  |
| **Ephrin receptor signaling** | **0.042** | **0.030** |  |  |  |  |  |  |  |  |  |  |
| **Sonic hedgehog signaling** | 0.044 |  |  |  |  |  |  |  |  |  |  |  |
| **Neurotrophin/TRK Signaling** |  | 0.006 |  | 0.001 |  |  |  |  |  |  |  |  |
| **Chondroitin Sulfate Biosynthesis** |  | 0.008 |  | 0.020 |  |  |  |  |  |  | 0.028 |  |
| **Pattern Recognition Receptors in Bacteria and Virus Recognition** |  | 0.013 |  | 0.004 |  |  |  |  |  |  |  |  |
| **Sphingolipid Metabolism** |  | 0.021 |  | 0.007 |  |  |  |  |  |  |  |  |
| **Macropinocytosis** |  | 0.027 |  |  |  |  |  |  |  |  |  |  |
| **Xenobiotic Metabolism Signaling** |  | 0.030 |  |  |  |  |  |  |  |  |  |  |
| **Parkinson's Signaling** |  | 0.032 |  | 0.018 |  |  |  |  |  |  |  |  |
| **Endoplasmic Reticulum Stress Pathway** |  | 0.040 | 0.0135 | 0.022 |  |  |  |  |  |  |  |  |
| **Glucocorticoid Receptor Signaling** |  |  |  | 0.041 |  |  |  |  |  |  |  |  |
| **Hepatic Cholestasis** |  |  |  |  |  | 0.040 |  |  |  |  |  |  |
| **Chemokine Signaling** |  |  |  |  |  |  |  | 0.005 |  |  |  |  |
| **GABA Receptor Signaling** |  |  |  |  |  |  |  | 0.010 |  |  |  |  |
| **Glycosphingolipid Biosynthesis - Ganglioseries** |  |  |  |  |  |  |  | 0.020 |  |  |  |  |
| **Glycosphingolipid Biosynthesis - Globoseries** |  |  |  |  |  |  |  | 0.020 |  |  |  |  |
| **Methionine Metabolism** |  |  |  |  |  |  |  | 0.022 |  |  |  |  |
| **Glycosphingolipid Biosynthesis - Neolactoseries** |  |  |  |  |  |  |  | 0.024 |  |  |  |  |
| **PI3K/AKT Signaling** |  |  |  |  |  |  |  | 0.024 |  |  |  |  |
| **Cardiac β-adrenergic Signaling** |  |  |  |  |  |  |  | 0.026 |  |  |  |  |
| **Dopamine Receptor Signaling** |  |  |  |  |  |  |  | 0.035 |  |  |  |  |
| **Actin Cytoskeleton Signaling** |  |  |  |  |  |  |  |  |  | 0.013 |  |  |
| **FGF Signaling** |  |  | 0.0437 |  |  |  |  |  |  | 0.016 |  |  |
| **PPARα/RXRα Activation** |  |  |  |  |  |  |  |  |  |  |  | 0.002 |
| **Starch and Sucrose Metabolism** |  |  |  |  | 0.039 |  |  |  |  |  |  | 0.009 |
| **Wnt/ β-catenin Signaling** |  |  |  |  |  |  |  |  |  |  |  | 0.027 |
| **RAR Activation** |  |  |  |  |  |  |  |  |  |  |  | 0.030 |
| **Aminosugars Metabolism** |  |  |  |  |  |  |  |  |  |  | 0.035 |  |
| **BMP signaling pathway** |  |  | 0.0063 |  | 0.023 |  |  |  |  |  |  |  |
| **Coagulation System** |  |  | 0.0004 |  | 0.0002 |  |  |  |  |  |  |  |
| **Cysteine Metabolism** |  |  |  |  |  |  |  |  |  |  | 0.031 |  |
| **G-Protein Coupled Receptor Signaling** |  |  |  |  |  |  |  |  |  |  | 0.025 |  |
| **Histidine Metabolism** |  |  |  |  |  |  |  |  | 0.001 |  | 0.026 |  |
| **Keratan Sulfate Biosynthesis** |  |  |  |  |  |  |  |  |  |  | 0.031 |  |
| **LPS/IL-1 Mediated Inhibition of RXR Function** |  |  |  |  |  |  |  |  |  |  | 0.025 |  |
| **Mitochondrial Dysfunction** |  |  |  |  |  |  | 0.042 |  |  |  |  |  |
| **Nicotinate and Nicotinamide Metabolism** |  |  |  |  | 0.039 |  |  |  |  |  |  |  |
| **O-Glycan Biosynthesis** |  |  | 0.0026 |  | 0.022 |  |  |  |  |  |  |  |
| **Pantothenate and CoA Biosynthesis** |  |  | 0.0182 |  | 0.013 |  |  |  |  |  |  |  |
| **Propanoate Metabolism** |  |  |  |  |  |  |  |  | 0.030 |  |  |  |
| **Pyruvate Metabolism** |  |  |  |  |  |  |  |  | 0.042 |  |  |  |
| **Riboflavin Metabolism** |  |  | 0.0166 |  | 0.012 |  |  |  |  |  |  |  |
| **Selenoamino Acid Metabolism** |  |  |  |  |  |  |  |  |  |  | 0.012 |  |
| **Sulfur Metabolism** |  |  |  |  |  |  |  |  |  |  | 0.005 |  |
| **TGF-β Signaling** |  |  | 0.0010 |  | 0.004 |  |  |  |  |  |  |  |
