# Supplementary material for: A Genome-Wide Investigation of SNPs and CNVs in Schizophrenia
Source: PLoS Genet. 2009 Feb 6;5(2):e1000373. doi: 10.1371/journal.pgen.1000373 (PMC2631150; doi:10.1371/journal.pgen.1000373)
Supplement: Text S1 — Supplementary methods and results. (0.09 MB PDF) [file pgen.1000373.s008.pdf]

A Genome-Wide Investigation of SNPs and CNVs in Schizophrenia: Supplementary Information

Supplementary Methods.

We took the following quality control steps to ensure that the genotypic data was accurate.

Infinium BeadStudio Raw Data Analysis

We brought all samples into a single BeadStudio file and evaluated the clustering of all samples using the standard Illumina cluster file. If a large number of samples did not fit the cluster for a random set of SNPs, we made a separate BeadStudio file for these samples. It was necessary to do this for the Munich samples. Once all files were made, we deleted any sample that had a very low intensity or a very low call rate using the Illumina cluster (<95%) . We then reclustered all SNPs that had a call frequency below 1, and deleted any sample that was below a 98% call rate after the reclustering. Next, we applied a "1% rule" so that all SNPs that had a call frequency below 99% were deleted. We also deleted any SNPs for which more than 1% of samples were not called or were ambiguously called. We have shown (unpublished data) that SNPs with many samples not called (or potentially miscalled) can lead to false positives in statistical associations.

We then screened the SNP data within BeadStudio using two criteria. First, we manually checked all SNPs with a cluster separation value below 0.3 to ensure correct calls. We were able to manually fix many of these SNPs, but some were deleted. Next, we evaluated any SNP (excluding X chromosome SNPs) with a Het Excess value between -1.0 to -0.1 and 0.1 to 1.0 to determine if the raw and normalized data show a clean call and deleted any SNP cluster that didn't appear "normal". This included SNPs that appear to show a deletion (hemizygotes and homozygous deletion). We did this because these are often artifacts from either the chemistry or an interfering SNP during hybridization. These procedures resulted in a 98.4% success rate of genotyping. We randomly selected one percent of DNA samples to be plated in duplicate for quality control checks. The concordance rate for duplicate genotyping was 99.997%. We also re-genotyped 5 SNPs on different chromosomes on the HumanHap550 chip using TaqMan assay in the Aberdeen samples. The concordance was 100%.

Minor allele frequency (MAF) check for data handling accuracy

We took this step to perform a basic check of the data accuracy on the data flow pipeline from the output of the Illumina genotyping facility to the analytical process. Using in-house software we

checked the MAF report from PLINK[1] against the original locus report generated by the genotyping facility. We assured that the two MAF reports matched exactly.

Specification of gender

In order to confirm the gender assignment obtained from the phenotype database, we used the observed genotypes of SNPs on chromosome X, and Y where available. We individually inspected all individuals who were marked as “male” but with significant amount of heterozygous X genotypes (>=1%), or who were marked as “female” but with high frequency of homozygous X genotypes (>=80%) or Y genotype readings using the original data source. If no satisfactory correction could be obtained, these individuals were excluded from further analyses. In total we excluded 3 Munich control participants, 5 Aberdeen cases, and 2 Aberdeen control participants in this step.

Cryptic relatedness

We took this step to check for unexpected relatedness between study participants. We estimated the sharing of genetic information by calculating identity by state (IBS) using the PLINK software. All pairs of DNA samples showing $\hat{\pi} \geq 0.125$ were individually inspected, and one sample in each pair was excluded from further analyses. In total, 2 Munich cases, 2 Munich controls, 4 Aberdeen cases, and 16 Aberdeen controls were removed in this step.

Skewed missingness

We took this step to determine whether the missing genotypes were skewed towards cases or controls and hence may give rise to spurious association p values. We used PLINK software to perform this check on the most associated SNPs and found no evidence of a missing data bias.

Hardy-Weinberg Equilibrium (HWE)

We performed this check using PLINK software on the most associated SNPs, separately in the Munich and Aberdeen cohorts. We defined a deviation from HWE with a criterion of p value less than 0.05 both in controls and in controls plus cases. No genotype data violated this check.

Recheck of the genotyping quality

We took this step as an individual recheck on the raw and normalized data for the most associated SNPs to be sure that they are called correctly as described in "*Infinium BeadStudio Raw Data Analysis*" process.

EIGENSTRAT outliers

We removed nine individuals (7 Munich cases, 1 Munich control, 1 Aberdeen case) because they were extreme outliers on one or more significant EIGENSTRAT axes.

Low MAF

We removed 939 SNPs with a MAF<0.0015. This criterion ensured that at least 6 individuals of the rare genotype were present in the combined Munich+Aberdeen dataset, to control for error in the estimation of asymptotic p values.

Curated EIGENSTRAT analyses

We selected EIGENSTRAT axes for use as covariates to adjust for ancestry in subsequent logistic regression analyses as follows. (1) To find EIGENSTRAT axes, we started with autosomal SNPs with MAF>0.01 from the "overlap" set of SNPs typed in both Munich and Aberdeen. (2) On inspection of SNP loadings for each PC axis (the "gamma" coefficients of Price et al. 2006 [2]), we found several of the top 10 axes to be dominated by a small number of SNPs all mapping to the same region of the genome. For example, PC axis 2 was found to be dominated by SNPs mapping to a region of chr8p22-23.1 coinciding with a known inversion polymorphism. (3) To correct for these LD effects, and ensure that EIGENSTRAT axes reflected only effects that applied equally across the whole genome (as ancestry effects should), we re-applied principal components analysis to a reduced SNP set in which (i) certain known high LD regions were excluded (chr8:8000000..12000000, chr6:25000000..33500000, chr11:45000000..57000000, chr5:44000000..51500000); (ii) SNPs were thinned using the "--indep-pairwise" option in PLINK software, such that all SNPs within a window size of 1500 (step size of 150) were required to have $r^2<0.2$; (iii) Each SNP was regressed on the previous 5 SNPs, and the residual entered into the PCA analysis, as suggested by Patterson et al (2006)[3]. (4) Inspection of SNP loadings on all axes deemed significant by the Tracy-Widom method of Patterson et al (2006) [3], using Q-Q plots against Normal expectation, now revealed no axes dominated by single high-LD regions of the genome. However, inspection of individual scores revealed 9 "outlier" individuals who exerted a large influence on one or more PC axes. These 9 individuals were removed and PCA reapplied to the

remaining dataset. (5) Tracy-Widom tests nominated the first 4 resulting PC axes as significant ($p<0.05$). In addition, schizophrenia case-control status associated significantly with PC axes 2-4, but no others in the first 10 axes. We therefore adopted the first four PC axes as covariates in subsequent analyses. PC axis 1 associated very clearly with division into Munich and Aberdeen samples (r=0.91) so to prevent multicollinearity the Munich/Aberdeen sampling location was not included as a covariate. Finally, we note that hidden population structure appears to be low in our data. We calculated the Devlin and Roeder inflation factor λ [4,5], adjusting for the known Munich/Aberdeen split, and obtained a value of 1.013 indicating that stratification was very slight and did not significantly affect the genetic associations we detected.

Comparison of methods for correcting multiple testing

We compared a simple Bonferroni correction against a randomization strategy for adjusting for multiple comparisons across correlated tests (here due to SNPs in LD). Since we wished to adjust for hidden stratification using EIGENSTRAT covariates, and since simple permutation of the raw y variate is known under certain conditions to lead to destabilized Type I error in the presence of covariates [6] we adopted the following randomization strategy. We first entered the y variate (here, case-control status for schizophrenia) into a logistic regression against the reduced model made up only of covariates (gender plus 4 EIGENSTRAT axes). We then created 1000 randomized datasets by drawing values for y from a Bernoulli distribution with probability derived from the fitted values of the reduced model logistic regression. Multiplicity-adjusted p-values were obtained from the proportion of randomized datasets containing a p value (for any SNP) equal to or less than the observed p value in question. We found that Bonferroni-adjusted values (found by multiplying the raw p value by the number of separate tests, here corresponding to 312,565 SNPs) resulted in adjusted p values that were always larger than the p values adjusted from the randomization strategy (as one would expect for positively-correlated tests), but always within a factor of two for the top hits in our dataset. We therefore found Bonferroni correction to be conservative, but acceptably so.

We provided a joint p value for the most associated SNPs, combined across all arms of the study. We used Stouffer's method [7] to combine p values from the genome-wide and replication stages, weighted according to sample size. We applied a Bonferroni correction for multiple testing, using all SNPs entered into the main part of Stage 1 analysis (n=312,565). Note that more SNPs were typed in the Aberdeen cohort, but these were not used in the combined analysis of Munich+Aberdeen data as they were not typed in the Munich cohort. The total of 312,565 SNPs is the correct number to use in

Bonferroni correction as in principle any of these SNPs could have been entered into the replication study under the Null hypothesis.

Primer/probe sequences for real time PCR: We used these primers and probes for real time PCR to quantify the four alternative transcripts of *ADAMTSL3*.
ASD-RSA transcript quantification:
forward: CCCTCCTGTGATAGTACGTACAC;
reverse: TTTTACAAACATACAGTAGTGAGTTGTGTCT;
probe: CTCCCAGGAGACTGC.

RSD-RSA transcript quantification:
forward: CTGGCGGCACTGTCTTG;
reverse: TTTTACAAACATACAGTAGTGAGTTGTGTCT;
probe: CCCTCCTGTGATAGAGAC.

RSD-ASA transcript quantification:
forward: CTGGCGGCACTGTCTTG;
reverse: CTGGCAGCACCTTTGTTTGTAG;
probe: CCCTCCTGTGATAACCG.

ASD-ASA transcript quantification:
forward: CCCTCCTGTGATAGTACGTACAC;
reverse: ACATGACTGGCAGCACCTT;
probe: CCAGACCGCTACAAACA.

The total *ADAMTSL3* mRNA expression was determined using a custom designed Taqman assay targeting exon 30: using forward primer CTCAAGTTGCAGGTTTTCAACAGTT, reverse primer GATCTATGAAAATGCCATTAATGCCAACA, probe CTGGCCAGAGCTTCTA, and using a commercially available Taqman assay for *ADAMTSL3* expression targeting the exon 17-18 boundary.

Genome-wide ExonArray Study

In a previously conducted study we assessed on a genome-wide scale how common genetic variation regulates the expression level of both whole transcripts and specific exons (as a measure of splicing) in

human cortical brain tissue collected from healthy control subjects[8]. In this study, the Affymetrix Human ST 1.0 Exon chips were used to assess exonic and transcript expression levels. Genome-wide genotyping was performed using the HumanHap 550 whole-genome chips. The association study, relating genetic variants included in the gene and the regions immediately surrounding it to the exon and the transcript level, was conducted using PLINK software (linear regression model) incorporating age, gender, postmortem interval (brain tissue samples), and processing days.

Supplementary Results

ADAMTSL3 variation controls alternative splicing

We first searched for alternative splicing events in the associated region (exons 28, 29, and 30) through the ExonHit Therapeutics splice array portal (http://portal.splicearray.com) and by blasting exon-intron boundary sequences against cDNA libraries. These analyses identified two potential sites for alternative splicing events involving IVS 29; (i) an alternative splice acceptor site (ASA) in exon 30 that is 63bp downstream of the reference splice acceptor (RSA) site, (ii) an alternative splice donor site (ASD) 17bp downstream of the reference donor splice site (RSD) in intron 29. These sites are predicted to generate four different transcripts (Figure S1A). The use of the ASD site generates an out-of-frame transcript and therefore no PLAC (protease and lacunin) domain (Figure S1B) while the use of the ASA site removes 21 amino acids from the PLAC domain (note, despite the presence of a PLAC domain, *ADAMTSL3* has no metalloprotease domain and is not predicted to have protease activity). The PLAC domain is itself of unknown function and is present in 20 confirmed proteins [9] (http://www.ebi.ac.uk/interpro/ISpy?ipr=IPR010909&tax=9606&mode=table&sort=ac&width=1280).

To assess whether genetic variation influences these alternative splicing events, we examined 30 samples of subjects with no known neuropsychiatric conditions and another 32 with a diagnosis of Alzheimer's Disease, from the Kathleen Price Bryan Alzheimer's Disease Brain Bank. We first confirmed the presence of all four alternative transcripts in human prefrontal and temporal cortex (Figure S1C). We next designed quantitative real time PCR assays to determine the abundance of each of the four alternative transcripts (Figure S2). We found that the rs950169 and rs2135551 polymorphisms show highly significant correlation with the use of the alternative splice acceptor site, resulting in a truncated PLAC domain. There is a four-fold increase in the RSD-ASA form in the minor-allele homozygotes compared with the major allele homozygotes ($p<.0001$, Figure S2), with a smaller effect on use of the ASD (Figure S3). Finally, we note that the effect of the polymorphisms on the absolute amount of the RSD-ASA is much higher than on the RSD-RSA transcript (the only

transcript form with a fully intact PLAC domain) suggesting the possibility that the truncating forms may represent a gain of function rather than reducing the amount of transcript encoding a protein with a functional PLAC domain.

Consistent with a role of these polymorphisms in splicing, rs950169 is located 10bp into exon 30 and the minor allele is predicted to increase binding of the SRp55 splicing factor (using the ESE finder software (http://rulai.cshl.edu/tools/ESE) [10], the C allele scores 2.91 and the T allele scores 5.16*)*, and is a primary candidate for mediating the observed splicing change.

Expression of *ADAMTSL3*

ADAMTSL3 is a widely-expressed glycoprotein of unknown function that contains immunoglobulin (Ig) and thrombospondin I domains with a postulated role in the extracellular matrix [11]. In the mouse, *Adamtsl3* mRNA is expressed in many brain regions (see the Allen Mouse Brain Atlas, http://www.brain-map.org), and, strikingly, is particularly high in the hippocampal formation, especially in the pyramidal cell layer of the CA1 and CA3 regions (Figure S4). This expression corresponds with the anatomical observation that schizophrenia is associated with both smaller pyramidal cells [12,13] and altered arborization in the CA1 and CA3 regions (though some references suggest that CA1 may be relatively spared [14]).

References

1. Purcell S, Neale B, Todd-Brown K, Thomas L, Ferreira MAR, et al. (2007) PLINK: a toolset for whole-genome association and population-based linkage analysis. Am J Hum Genet in press.
2. Price AL, Patterson NJ, Plenge RM, Weinblatt ME, Shadick NA, et al. (2006) Principal components analysis corrects for stratification in genome-wide association studies. Nat Genet 38: 904-909.
3. Patterson N, Price AL, Reich D (2006) Population structure and eigenanalysis. PLoS Genet 2: e190.
4. Devlin B, Roeder K (1999) Genomic control for association studies. Biometrics 55: 997-1004.
5. Reich DE, Goldstein DB (2001) Detecting association in a case-control study while correcting for population stratification. Genet Epidemiol 20: 4-16.
6. Anderson MJ, Legendre P (1999) An empirical comparison of permutation methods for tests of partial regression coefficients in a linear model. J stat comput simul 62: 271-303.
7. Whitlock MC (2005) Combining probability from independent tests: the weighted Z-method is superior to Fisher's approach. J Evol Biol 18: 1368-1373.
8. Heinzen EL, Ge, D.,Cronin, K.D., Maia, J.M., Shianna, K.V., Gabriel, W.N., Welsh-Bomer, K.A., Hulette., C.M., Ddenny, T.N., Goldstein, D.B. (2008) Tissue-specific genetic control of splicing: Implications for the study of complex traits. PLoS Biol.
9. Mulder NJ, Apweiler R, Attwood TK, Bairoch A, Bateman A, et al. (2007) New developments in the InterPro database. Nucleic Acids Res 35: D224-228.
10. Cartegni L, Wang J, Zhu Z, Zhang MQ, Krainer AR (2003) ESEfinder: A web resource to identify exonic splicing enhancers. Nucleic Acids Res 31: 3568-3571.
11. Hall NG, Klenotic P, Anand-Apte B, Apte SS (2003) ADAMTSL-3/punctin-2, a novel glycoprotein in extracellular matrix related to the ADAMTS family of metalloproteases. Matrix Biol 22: 501-510.
12. Liu L, Schulz SC, Lee S, Reutiman TJ, Fatemi SH (2007) Hippocampal CA1 Pyramidal Cell Size is Reduced in Bipolar Disorder. Cell Mol Neurobiol.
13. Rosoklija G, Toomayan G, Ellis SP, Keilp J, Mann JJ, et al. (2000) Structural abnormalities of subicular dendrites in subjects with schizophrenia and mood disorders: preliminary findings. Arch Gen Psychiatry 57: 349-356.
14. Harrison PJ (2004) The hippocampus in schizophrenia: a review of the neuropathological evidence and its pathophysiological implications. Psychopharmacology (Berl) 174: 151-162.
